# New discoveries in the field of metabolism by applying single-cell and spatial omics

**DOI:** 10.1016/j.jpha.2023.06.002

**Published:** 2023-06-04

**Authors:** Baocai Xie, Dengfeng Gao, Biqiang Zhou, Shi Chen, Lianrong Wang

**Affiliations:** a Department of Critical Care Medicine, Shenzhen Institute of Translational Medicine, Shenzhen Second People's Hospital, The First Affiliated Hospital of Shenzhen University, Guangdong Key Laboratory for Biomedical Measurements and Ultrasound Imaging, National-Regional Key Technology Engineering Laboratory for Medical Ultrasound, School of Biomedical Engineering, Shenzhen University Medical School, Shenzhen, Guangdong, 518060, China; b Department of Respiratory Diseases, The Research and Application Center of Precision Medicine, The Second Affiliated Hospital of Zhengzhou University, Zhengzhou University, Zhengzhou, 450014, China; c State Key Laboratory of Animal Biotech Breeding, College of Biological Sciences, China Agricultural University, Beijing, 100193, China; d Department of Geriatric & Spinal Pain Multi-Department Treatment, Shenzhen Second People's Hospital, The First Affiliated Hospital of Shenzhen University, Health Science Center, Shenzhen, Guangdong, 518035, China; e Department of Gastroenterology, Ministry of Education Key Laboratory of Combinatorial Biosynthesis and Drug Discovery, Zhongnan Hospital of Wuhan University, School of Pharmaceutical Sciences, Wuhan University, Wuhan, 430071, China

**Keywords:** SCM-Omics, SM-Omics, Obesity, Diabetes, NAFLD, CVD

## Abstract

Single-cell multi-Omics (SCM-Omics) and spatial multi-Omics (SM-Omics) technologies provide state-of-the-art methods for exploring the composition and function of cell types in tissues/organs. Since its emergence in 2009, single-cell RNA sequencing (scRNA-seq) has yielded many groundbreaking new discoveries. The combination of this method with the emergence and development of SM-Omics techniques has been a pioneering strategy in neuroscience, developmental biology, and cancer research, especially for assessing tumor heterogeneity and T-cell infiltration. In recent years, the application of these methods in the study of metabolic diseases has also increased. The emerging SCM-Omics and SM-Omics approaches allow the molecular and spatial analysis of cells to explore regulatory states and determine cell fate, and thus provide promising tools for unraveling heterogeneous metabolic processes and making them amenable to intervention. Here, we review the evolution of SCM-Omics and SM-Omics technologies, and describe the progress in the application of SCM-Omics and SM-Omics in metabolism-related diseases, including obesity, diabetes, nonalcoholic fatty liver disease (NAFLD) and cardiovascular disease (CVD). We also conclude that the application of SCM-Omics and SM-Omics approaches can help resolve the molecular mechanisms underlying the pathogenesis of metabolic diseases in the body and facilitate therapeutic measures for metabolism-related diseases. This review concludes with an overview of the current status of this emerging field and the outlook for its future.

## Highlights

• Single-cell Multi-Omics (SCM-Omics) and Spatial Multi-Omics (SM-Omics) technologies have been applied to achieve breakthroughs in metabolic disease research.
• We provide an outline of the development and history of SCM-Omics and SM-Omics technologies.
• We review the application of SCM-Omics and SM-Omics in metabolism-related diseases, including obesity, diabetes, nonalcoholic fatty liver disease (NAFLD), and cardiovascular disease (CVD).
• We also conclude that applying SCM-Omics and SM-Omics methods can enhance our understanding of metabolic disease pathogenesis in vivo and facilitate therapeutic measures.

## Graphical abstract

Single-cell Multi-Omics (SCM-Omics) and Spatial Multi-Omics (SM-Omics) technologies provide state-of-the-art methods for exploring the composition and function of cell types in tissues/organs. Here, we review the evolution of SCM-Omics and SM-Omics technologies, as well as the application progress of SCM-Omics and SM-Omics in metabolism-related diseases, including obesity, diabetes, nonalcoholic fatty liver disease (NAFLD) and cardiovascular disease (CVD). We also conclude that applying SCM-Omics and SM-Omics approaches can help resolve the molecular mechanisms underlying the pathogenesis of metabolic diseases in the body and facilitate therapeutic measures for metabolic and metabolism-related diseases. This review concludes with an overview of the current status of this emerging field and our outlook for its future.

**Figure:**
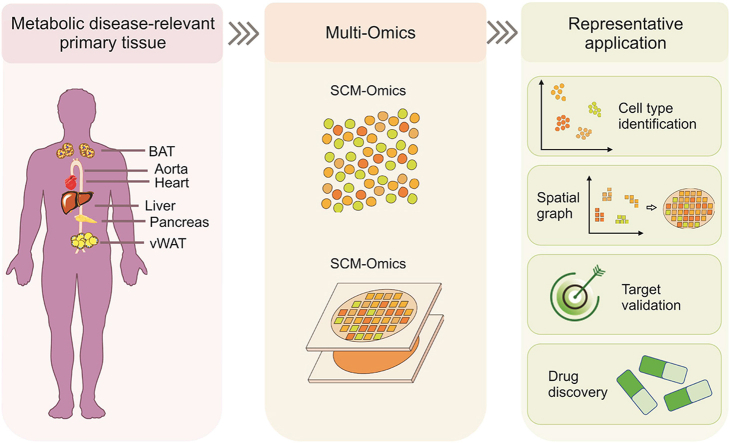

## 1 Introduction

Cells are the basic unit of biological, morphological, structural, and functional activities and the basis of life activities. Since the advent of single-cell transcriptome sequencing technology in 2009 [1], new avenues have opened for the study of molecular mechanisms in single cells. The release of the Human Cell Atlas Project in 2017 ushered in a new era of single-cell sequencing [2]. Single-cell multi-Omics (SCM-Omics) was named the Technology of the Year by Nature Methods in 2019 [3], and spatial transcriptome technology was named the Technology of the Year in 2020 [4]. At present, SCM-Omics and spatial multi-Omics (SM-Omics) technologies allow us to not only precisely understand the expression of molecules within each cell but also map the spatial location of that cell and the expression of genes in that spatial location [5,6]. With the advent of spatiotemporal omics technology [7], the distribution of cells and interactions between cells during the development of metabolic diseases can be presented at the cellular or even subcellular level, enabling truly high-precision structural insight. The application of these new technologies to analyze the molecular mechanisms of metabolic disease development has contributed to our overall understanding of these diseases, which is of great significance to the realization of precision medicine in the future.

SCM-Omics and SM-Omics techniques have been applied innovatively in neuroscience, developmental biology, and cancer research [8], particularly for assessing tumor heterogeneity and T-cell infiltration [9]. In recent years, the use of these techniques in research on metabolic diseases, including obesity [10], diabetes [11], nonalcoholic fatty liver disease (NAFLD) [12], and cardiovascular disease (CVD) [13] have increased. With the advancement of SCM-Omics and SM-Omics technology, SCM-Omics technology can use genetic information, epigenetic information, transcriptome information, and metabolome information of a single cell to help us obtain more in-depth information on the characteristics and regulatory mechanisms of cells. With respect to the diagnosis of metabolic diseases, SCM-Omics technology can help us identify different types of cells, detect changes in metabolic pathways and find new diagnostic markers [14]. SM-Omics technology can help us discover the molecular mechanisms of metabolic diseases and the spatial variation of disease molecules. We can identify the intermediate products of metabolite degradation using SM-Omics technology to further explore the byproducts of metabolite degradation and their possible adverse effects on the human body [15,16]. In summary, SCM-Omics and SM-Omics technologies can help us better understand the molecular mechanisms of metabolic diseases. However, the application of SCM-Omics and SM-Omics technology on metabolic diseases is still subject to many limitations. More basic research is needed to ensure the reliability and accuracy of these techniques. Many challenges and issues need to be faced, including the processes of standardization and standardized data exchange, the reliability of the technology, and other issues such as algorithm optimization and data analysis. With the continuous improvement and development of related technologies, these problems will be gradually solved, and the relevant research results will be further improved. The application of SCM-Omics and SM-Omics will also further advance our understanding of metabolism-related diseases.

SCM-Omics and SM-Omics technologies are developing rapidly and are widely used in various fields of life sciences. Moreover, the advancement of big data computing capabilities and the updating of multi-omics joint analysis algorithms have yielded more complete information. The application of these technologies has allowed us to gain a more in-depth understanding of the specific mechanisms underlying the development of metabolic diseases and to screen for targets of action for the treatment of metabolic diseases. Thus, the development of drugs for metabolic diseases has accelerated. In addition to dissecting the pathogenesis of diseases, SCM-Omics and SM-Omics can contribute to clinical precision medicine. The effects of treatments for metabolic diseases have differences, which may be related to individual differences. The application of SCM-Omics and SM-Omics enables assessment of individual cell type changes and the functional status and thus improves the accuracy of our understanding of each metabolic disease using the existing diagnostic tools. It is expected that this technology will be used in future clinical practice and for the development of personalized medicine to prevent or treat metabolic diseases. Here, we review the evolution of SCM-Omics and SM-Omics technologies, and then describe the progress in the application of SCM-Omics and SM-Omics in metabolism-related diseases (including obesity, diabetes, NAFLD, and CVD) specifically, and clarify that the application of SCM-Omics and SM-Omics methods can tap into new molecular mechanisms of metabolic disease occurrence and provide new methods and ideas to promote the prevention and treatment of metabolism-related diseases. It is also clear that applying SCM-Omics and SM-Omics can uncover new molecular mechanisms of metabolism-related diseases, and provide new approaches and ideas to promote the prevention and treatment of metabolism and metabolism-related diseases. The current status of research in this emerging field and its prospects for future applications are then outlined.

## 2 The evolution of SCM-Omics and SM-Omics

**Fig. 1:**
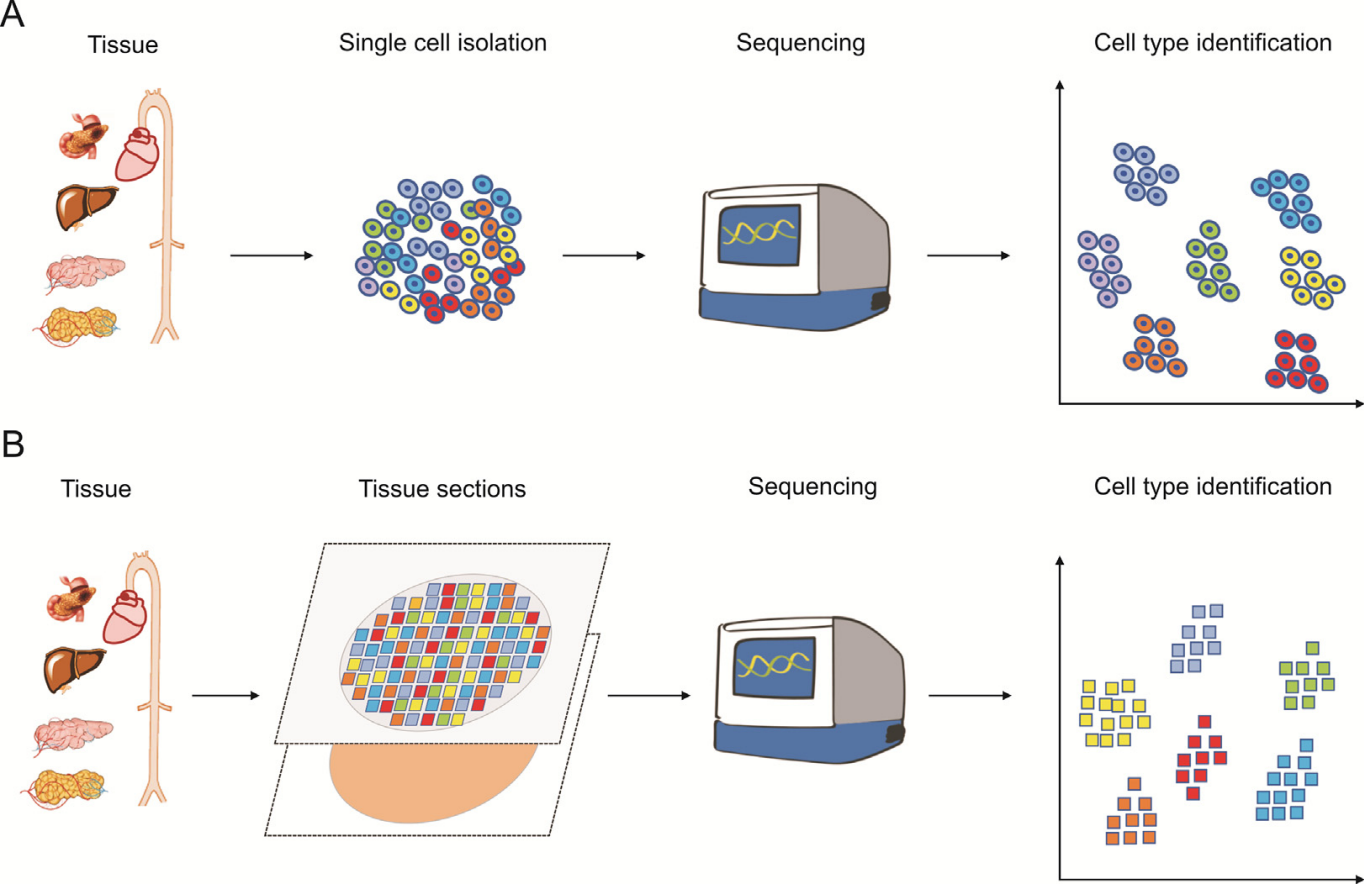
Single-cell multi-Omics (SCM-Omics) and spatial multi-Omics (SM-Omics) techniques. (A) Brief schematic diagram of the SCM-Omics techniques process. (B) Brief schematic diagram of the SM-Omics techniques process.

SCM-Omics and SM-Omics technologies are currently the most popular technologies in the fields of bioinformatics and life sciences. SCM-Omics technology is a technique that can detect different cell types in a tissue at the same time. Typically, when using this technique, the tissue is dissected into individual cells, and then the cells are sequenced or analyzed to determine the cell types and gene expression patterns in the tissue [17]. This technology plays an important role in improving our understanding of the composition of complex cell types in organisms and their corresponding cellular functions (Fig. 1A). SM-Omics technology is a method that combines the structural orientation of cells and tissues, and can be used to simultaneously analyze different molecular markers in tissues and infer location relationships at the cellular scale [6]. This technology can locate changes in gene expression patterns, cell signaling pathways, and other key factors in three-dimensional (3D) space within cells [18]. The development of this technology enables scientists to study the guidance and distribution patterns of molecules in biological organisms through detailed imaging at the cellular level, especially in the analysis of complex and diverse tissue environments (Fig. 1B). These technologies enable scientists to explore the differences between different tissues in organisms, understand cell types, gene expression patterns, and corresponding biological functions at the cellular level.

**Fig. 2:**
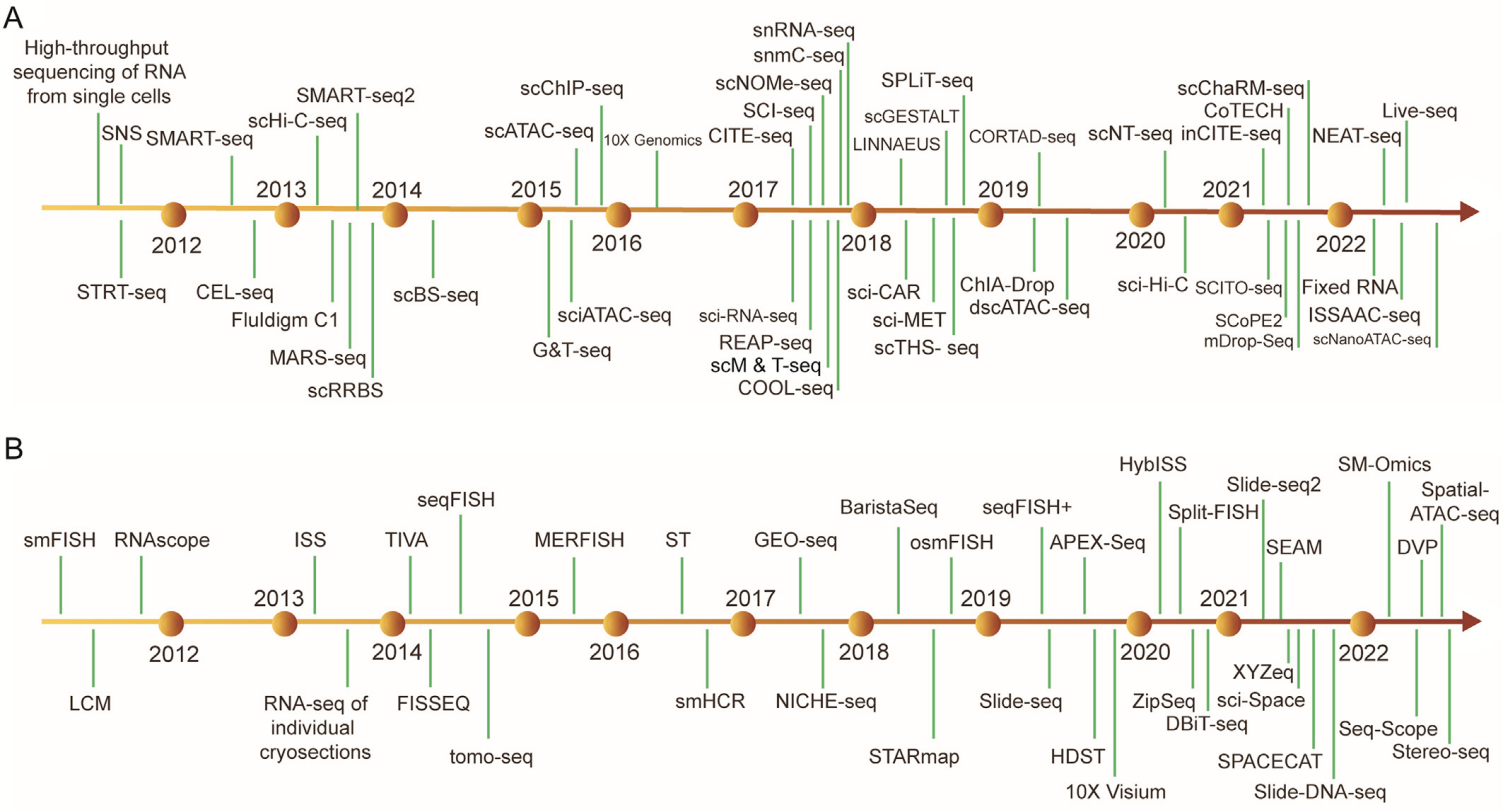
Timeline of single-cell multi-Omics (SCM-Omics) and spatial multi-Omics (SM-Omics) method milestones. (A) Timeline of publications on single-cell omics approaches. (B) Timeline of publications on spatial omics approaches. STRT-seq: single-cell tagged reverse transcription sequencing; SNS: single-nucleus sequencing; SMART-seq: full-transcriptome mRNA-seq; CEL-seq: cell expression by linear amplification and sequencing; scHiC-seq: single-cell Hi-C; Fluidigm C1: the C1 system isolates single cells into individual reaction chambers in the exclusive Fluidigm integrated fluidic circuit; MARS-seq: massively parallel RNA single-cell sequencing framework; scRRBS: single-cell reduced-representation bisulfite sequencing; scBS-seq: single-cell bisulfite sequencing; G&T-seq: genome & transcriptome sequencing; sciATAC-seq: single-cell combinatorial indexing ATAC-seq; scATAC-seq: single-cell ATAC-seq; scChIP-seq: single-cell ChIP-seq; sci-RNA-seq: single cell combinatorial indexing RNA sequencing; CITE-seq: cellular indexing of transcriptomes and epitopes; SCI-seq: single-cell combinatorial indexed sequencing; scNOME-seq: single-cell nucleosome occupancy and methylome sequencing; snmC-seq: single nucleus methylcytosine sequencing; snRNA-seq: single nucleus RNA sequencing; REAP-seq: RNA expression and protein sequencing assay; scM&T-seq: a method for parallel single-cell genome-wide methylome and transcriptome sequencing; COOL-seq: single-cell chromatin overall omic-scale landscape sequencing; LINNAEUS: lineage tracing by nuclease-activated editing of ubiquitous sequences; sci-CAR: a combinatorial indexing-based co-assay that jointly profiles chromatin accessibility and mRNA in each of thousands of single cells; sci-MET: single-cell combinatorial indexing for methylation analysis; scTHS-seq: single cell transposome hypersensitive sites sequencing; scGESTALT: a method that combines cumulative editing of a lineage barcode array by CRISPR–Cas9 with large-scale transcriptional profiling using droplet-based single-cell RNA sequencing; SPLiT-seq: split-pool ligation-based transcriptome sequencing; ChIA-Drop: a strategy for multiplex chromatin-interaction analysis via droplet-based and barcode-linked sequencing; CORTAD-seq: a method for concurrent sequencing of the transcriptome and targeted genomic regions; dscATAC-seq: droplet single-cell assay for transposase-accessible chromatin using sequencing; scNT-seq: single-cell metabolically labeled new RNA tagging sequencing; sci-Hi-C: single-cell combinatorial indexed Hi-C; inCITE-seq: intranuclear CITE-seq; CoTECH: combined assay of transcriptome and enriched chromatin binding; scChaRM-seq: single-cell chromatin accessibility, RNA barcoding, and DNA methylation sequencing; SCITO-seq: single-cell combinatorial indexed cytometry sequencing; SCoPE2: single cell proteomics; mDrop-seq: single cell RNA-seq of microbes; NEAT-seq: simultaneous profiling of intranuclear proteins, chromatin accessibility and gene expression in single cells; Fixed RNA: the single cell Fixed RNA Profiling (FRP) workflow measures RNA levels in samples (single cells or nuclei) fixed with formaldehyde, using probes targeting the whole transcriptome; ISSAAC-seq: in situ sequencing hetero RNA–DNA-hybrid after assay for transposase-accessible chromatin-sequencing; Live-seq: a technology that keeps the cell alive after transcriptome profiling by using a cytoplasmic biopsy; scNanoATAC-seq: single-cell nanowell-assisted assay for transposase-accessible chromatin using sequencing. smFISH: amplification-based single molecule fluorescence in situ hybridization; RNAscope: a commercially available in situ hybridization assay for the detection of RNA in formalin-fixed paraffin-embedded tissue; LCM: laser capture microdissection; ISS: in situ sequencing; TIVA: transcriptome in vivo analysis; FISSEQ: fluorescent in situ sequencing; sequential fluorescence in situ hybridization; seqFISH: sequential fluorescence in situ hybridization; tomo-seq: RNA-seq tomography; MERFISH: multiplexed error-robust fluorescence in situ hybridization; ST: spatial transcriptomics; smHCR: single-molecule hybridization chain reaction; GEO-seq: geographical position sequencing; NICHE-seq: an optimization of a targeted, padlock probe-based technique for in situ barcode sequencing compatible with Illumina sequencing chemistry; BaristaSeq: an optimization of a targeted, padlock probe-based technique for in situ barcode sequencing compatible with Illumina sequencing chemistry; STARmap: spatially-resolved transcript amplicon readout mapping; osmFISH: ouroboros smFISH; seqFISH+: evolution of sequential fluorescence in situ hybridization; Slide-seq: a scalable technology for measuring genome-wide expression at high spatial resolution; APEX-seq: a method for RNA sequencing based on direct proximity labeling of RNA using the peroxidase enzyme APEX2; HDST: high-definition spatial transcriptomics; 10X Visium: Visium Spatial Gene Expression; HybISS: hybridization-based in situ sequencing; Split-FISH: a multiplexed fluorescence in situ hybridization method; ZipSeq: a method that uses patterned illumination and photocaged oligonucleotides to serially print barcodes (‘zipcodes’) onto live cells in intact tissues, in real time and with an on-the-fly selection of patterns; DBiT-seq: deterministic barcoding in tissue for spatial omics sequencing; XYZeq: a workflow that encodes spatial metadata into scRNA-seq libraries; sci-Space: a method that retains single cell resolution while resolving spatial heterogeneity at larger scales; SEAM: spatial single nuclear metabolomics; SPACECAT: spatially photo activatable color encoded cell address tags; Slide-DNA-seq: a method for capturing spatially resolved DNA sequences from intact tissue sections; SM-Omics: spatial multi-omics; Seq-Scope: a spatial barcoding technology with a resolution comparable to an optical microscope; DVP: deep visual proteomics; Spatial-ATAC-seq: spatially resolved assay for transposase-accessible chromatin using sequencing; Stereo-seq: spatial enhanced resolution omics sequencing.

In the past few decades, SCM-Omics technology has been rapidly developing. Early single-cell studies mainly focused on the morphological and growth characteristics of individual cells. However, due to technical limitations, few studies have investigated biological properties such as gene expression and metabolite composition in individual cells. With the development of high-throughput technologies such as gene chips, RNA sequencing, and metabolite analysis, SM-Omics technology has made rapid progress. Tang et al. [1] reported a single-cell mRNA transcriptome sequencing technique that optimized the mRNA sequencing method proposed in 2009, and this new technique made it possible to determine transcriptome information of a single cell. This technique ushered in a new era of single-cell analysis. With the rapid development of single-cell transcriptomics technology, researchers have realized that proteomics and metabolomics also require higher resolution solutions, leading to the subsequent appearance of single-cell proteomics and metabolomics technologies. Stoeckius et al. [19] reported a technique based on single-cell transcriptomics and high-throughput protein analysis in 2014, and this technique allowed the first joint analysis of RNA and protein in the same cell and opened up a new era of single-cell proteomics. Zhang et al. [20] reported a microfluidic chip in 2016 that can simultaneously measure gene expression and metabolite concentration in single cells, marking a new breakthrough in single-cell metabolomics. Subsequent developments in microfluidic technology, high-throughput methods, and related algorithms have continuously updated and improved SCM-Omics technology [[21], [22], [23], [24], [25], [26], [27], [28], [29], [30], [31], [32], [33], [34], [35], [36], [37]] (Fig. 2A). For example, Haimovich et al. [38] reported a technique called single molecule fluorescence in situ hybridization-flow cytometry (smFISH-Flow) in 2018, which can simultaneously measure cell epigenetics and transcriptomics information. It combines the image information of epigenetics with the high-throughput nature of flow cytometry, which enables high-throughput multi-omics analysis at the single-cell level. In the same year, Butler et al. [39] proposed a complementary learning framework that combines flow cytometry and RNA sequencing, developing a cell analysis technique that does not require sorting, further expanding the depth and breadth of single-cell analysis.

SM-Omics refers to the application of multiple technologies and methods to the same set of sample data, and this technique integrates the results from these methods to comprehensively and accurately describe the complex molecular characteristics and physiological processes within an organism. This technology can be traced back to the analysis of ribosomal RNA (rRNA) with in situ hybridization (ISH) in 1969 [40]. From the last century to the early 21st century, the origination of SM-Omics was mainly related to exploring new technologies and establishing methodologies, primarily based on chemometrics, and thus revealing the spatial distribution of various molecular components through processing spectral images [41,42]. From 2005 to 2010, SM-Omics technology continued to develop, and new technological breakthroughs constantly emerged. The combination of flow cytometry and imaging technology achieved high-throughput single-cell analysis, opening a new chapter in cell heterogeneity analysis [[43], [44], [45]]. From 2010 to 2015, precision technology became an essential direction for the development of SM-Omics. High-throughput detection technology based on microRNAs can rapidly and accurately detect various microRNAs in biological samples [46,47], achieving accurate analysis at the nucleic acid level. At the same time, the emergence of a new spatial transcriptomics technique called single-cell RNA sequencing (scRNA-seq) enables researchers to obtain gene expression profiles of individual cells, better understand cell heterogeneity under different cell types, developmental stages, and pathological states [48,49]. From 2015 to now, the development of next-generation high-throughput sequencing technology has provided more vital technical support for SM-Omics research [[50], [51], [52], [53], [54], [55], [56], [57], [58], [59], [60], [61], [62], [63], [64], [65], [66], [67], [68], [69], [70], [71], [72], [73], [74], [75]] (Fig. 2B). The emergence of nanopore single-molecule sequencing technology has higher accuracy, speed, and detection depth than traditional sequencing methods, and thus provides more accurate data analysis for SM-Omics research [[76], [77], [78]]. Moreover, SM-Omics analysis platforms based on artificial intelligence and machine learning algorithms continue to emerge, significantly improving the efficiency and accuracy of data processing and analysis [79,80]. Overall, SM-Omics technology is undergoing rapid development and constantly emerging with many landmark technological breakthroughs, providing broader research space for life science research.

In metabolic disease research, the application of SCM-Omics and SM-Omics technologies has demonstrated their enormous value. These multi-omics technologies can provide new methods and diagnostic tools for metabolic disease research. Through the use of these technologies, it is possible to understand metabolic diseases at the molecular and cellular levels. By combining the use of SCM-Omics and SM-Omics technologies, scientists can better understand the molecular mechanisms in cells and tissues, identify specific changes, and inspire new methods for drug discovery. For example, using SCM-Omics technology, researchers can gain a deeper understanding of the impact of drugs on specific cells, and can thus develop more scientific drug development plans [81,82]. SM-Omics technology also plays an essential role in the clinical diagnosis of diseases. Scientists can use this technology to clarify metabolic disease diagnosis, classify metabolic disease symptoms, and develop more accurate diagnostic methods [83,84]. In summary, the new opportunities and challenges brought by SCM-Omics and SM-Omics technologies will play an essential role in promoting the development and prevention of metabolic diseases in medicine.

## 3 The application of SCM-Omics and SM-Omics in obesity

Obesity as a public health problem has become more severe in recent years [85]. Exploring the pathogenesis and treatment of obesity is of great importance for improving human health. Usually, when the body is in a state where energy intake exceeds energy expenditure, adipose tissue expands. This expansion is evidenced by an increase in the number and volume of adipocytes and changes in the number of adipose vascular stromal cells. These changes affect the regulation of energy balance in the body. The expansion of adipose tissue depends on two main aspects. The first aspect is the proliferation and differentiation of adipocyte progenitor cells, which have been shown to prevent obesity and related diseases [86]. The second aspect is the hypertrophy of mature adipocytes, which is associated with metabolic disturbances caused by obesity-induced insulin resistance [87]. Therefore, the expansion of adipose tissue plays an essential role in the development of metabolic diseases. However, little is known about the specific mechanisms by which adipocytes cause disease. Previous studies on adipocyte differentiation usually used the stromal vascular fractions (SVF) or stromal-vascular cells and adipose-derived mesenchymal stem cells, which are highly heterogeneous cell populations [88]. This is also the basis for further research on obesity [89]. Therefore, applying SCM-Omics and SM-Omics techniques to gain insight into the specific changes in cell types in adipose tissue during obesity development has an irreplaceable role in deciphering the molecular mechanisms involved in adipose tissue expansion in obesity and its related metabolic diseases.

**Fig. 3:**
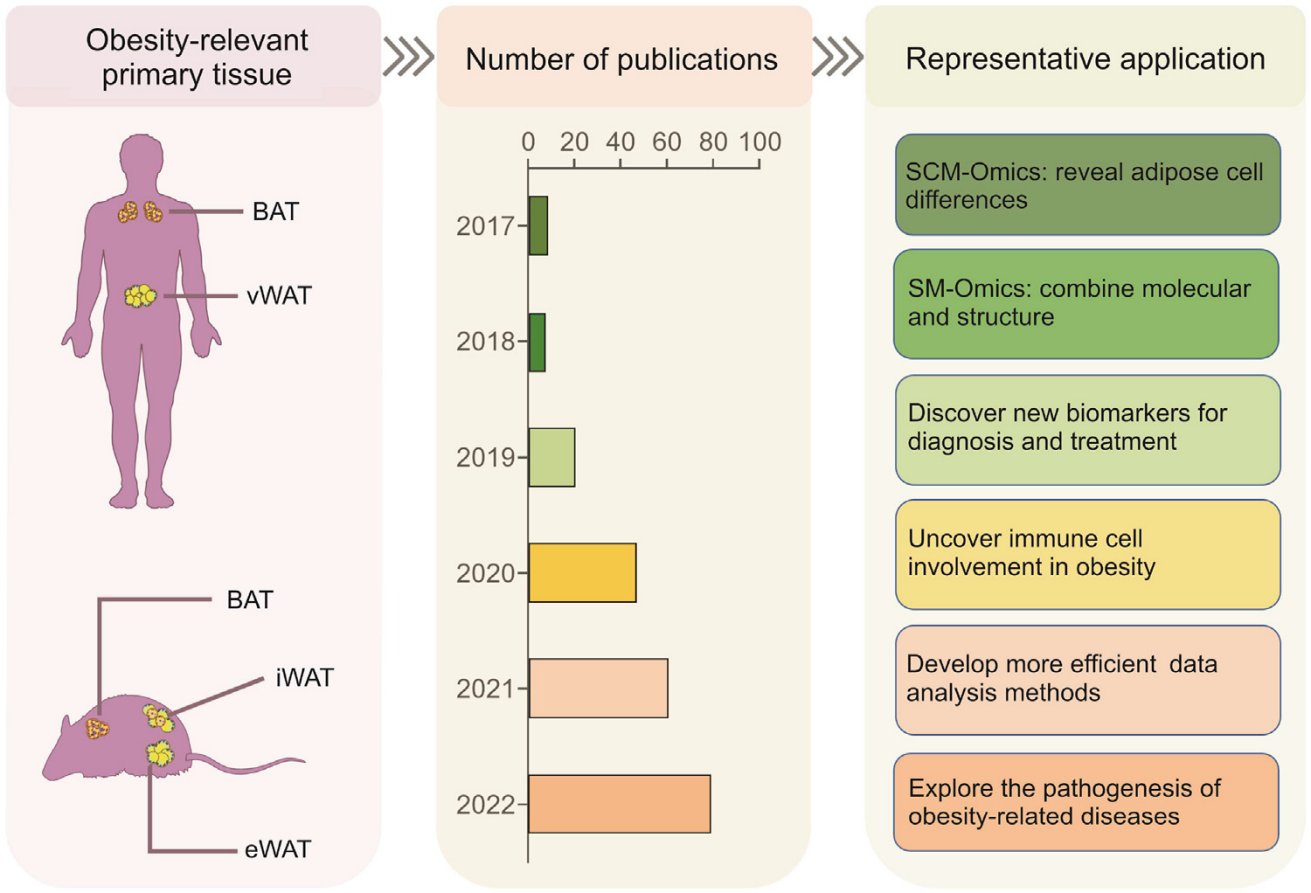
Application of single-cell multi-Omics (SCM-Omics) and spatial multi-Omics (SM-Omics) technologies in obesity research. The application of SCM-Omics and SM-Omics technologies in obesity-relevant primary tissues in humans and mice, namely brown adipose tissue (BAT), visceral white adipose tissue (vWAT), inguinal white adipose tissue (iWAT), and epididymal white adipose tissue (eWAT), is described. The timeline shows the number of publications on SCM-Omics and SM-Omics techniques in the PubMed database over the past six years (2017–2022). Representative applications in obesity research in humans and mice are described through publications related to SCM-Omics and SM-Omics technologies.

With the advent of SCM-Omics and SM-Omics technologies in recent years, it has become possible to determine cellular heterogeneity and function at the single-cell level [90]. Current high-throughput microfluidic technologies can capture thousands of cells from each sample for simultaneous sequencing and clustering with continuously updated algorithms and visual modeling software. High-throughput sequencing is performed using disease-associated tissue samples to efficiently analyze the cellular composition and the risk of disease action. Recent studies applying scRNA-seq to the analysis of adipose tissue from different sites in humans and mice have yielded some outstanding results (Fig. 3). For example, a subpopulation of adipocyte progenitors that regulates adipocyte differentiation was identified [91], and a new type of inflammatory progenitor cells inhabiting the visceral fat in mice was revealed [92]. In addition, a novel adipocyte-derived cell, the dipeptidyl peptidase 4 positive (DPP4^+^) subpopulation of cells, has been identified, mainly in the mesenchyme [93]. The mesenchymal tissue is located in the subcutaneous tissue and various organ tissues and is interconnected with fluid-filled reticular structures, which are likely the largest organs of the body. Therefore, these findings obtained by applying scRNA-seq techniques open new directions for exploring the relationship between adipose tissue and metabolic diseases.

Here, we introduce the latest research on the application of SCM-Omics and SM-Omics technologies in obesity (Table S1), including the current sequencing methods: scRNA-seq [[91], [92], [93], [94], [95], [96], [97], [98], [99], [100]], single-nuclei RNA sequencing (snRNA-seq) [97,[101], [102], [103]], full-length scRNA-seq/snRNA-seq [104], and spatial transcription sequencing [105]. The current main application platforms, such as 10x Genomics and the Fluidigm C1 system, are considered, and the sample size, the number of cells captured, and the number of tissue-covered spots for each study are discussed. Furthermore, the main contributions of each article are summarized. These articles show the multifaceted nature of adipose tissue from different research directions, and we divide them into different groups according to their different research directions. For example, a similar strategy was used in a human study, in which a scRNA-seq cluster analysis of 26,350 previous studies on adipocyte cells captured from 25 fat samples identified 17 types [95]. Sun et al. [101] used scRNA-seq to identify a subpopulation of cells that regulate thermogenesis in mouse adipose tissue and found that human adipose tissue contains more of this type of cell. These findings suggest that targeting these cells could potentially restore thermogenic activity. Furthermore, scRNA-seq can answer questions that were previously unanswered, such as those related to diet-induced obesity. scRNA-seq technology has been applied to reveal the plasticity of adipose tissue [103]. Inflammation in the epididymal white adipose tissue (eWAT) of obese mice has been found to affect not only localized phenomena near the coronary structures but also a broader tissue region. These studies provide data that lay the foundation for future studies of adipocyte differentiation and adipose tissue plasticity.

In conclusion, SCM-Omics techniques have enabled the integrated analysis of nonadipocyte and in situ information on adipose tissue. Recent research has focused on humans and mice [[91], [92], [93], [94], [95], [96], [97], [98], [99], [100]]. In the future, more animal models can be utilized to clarify the differences in the type and function of adipose tissue between species. Due to the technical limitations of previous scRNA-seq techniques, which removed this lipid-rich fraction in the process, many studies were performed only with SVF cells. However, this approach does not consider the adipocyte transcriptional profile. Single-cell transcriptional analysis can now be achieved by extracting nuclei from adipose tissue or cells, namely, snRNA-seq analysis [[101], [102], [103]]. In addition, spatial transcriptome analysis of adipose tissue is now possible, and thus, the recovery of information on the location of adipose tissue can be considered [105]. This allows researchers to better understand the spatial distribution pattern of obesity lesions and even to determine the extent to which specific cell types contribute to the lesions. With this technique, researchers can generate highly accurate three-dimensional structural images, making single-cell sequencing data more meaningful and leading to a better understanding of the complexity of obesity markers and pathogenic mechanisms. Moreover, combining SCM-Omics analysis with SM-Omics analysis will provide us with more valuable findings in the future as bioinformatics technology advances, which would enrich the current knowledge of the role of adipose tissue in systemic metabolic regulation. In addition, many valuable results have been obtained through studies in human and mouse adipose tissue, and many cellular taxa or target genes related to metabolic health have been identified. Therefore, further research is needed to determine these targets as metabolic functions in the future. This would increase the feasibility of treating such disorders and improve the development of their corresponding therapeutic drugs. In summary, SCM-Omics and SM-Omics technologies have strong application prospects in obesity research. They are expected to solve multiple issues in obesity research, including identifying the etiology, determining pathological features, exploring the mechanisms of disease progression, and discovering new therapies and treatment strategies. The application of these technologies will bring more accurate and in-depth research results and provide crucial fundamental support for future studies on treating obesity.

## 4 The application of SCM-Omics and SM-Omics in diabetes

Diabetes is one of the major diseases that seriously threatens human life and health [106]. A recent report from the International Diabetes Federation suggests that approximately 415 million individuals have diabetes worldwide, and more than 90% of these individuals have type 2 diabetes (T2D) [107]. Despite the growing awareness of diabetes risk factors and the development of prevention programs, its incidence and prevalence continue to rise globally [108]. Both type 1 diabetes (T1D) and T2D are related to the function of the pancreas. The cause of T1D is dysfunction of the pancreas, resulting in the production of little or no insulin. The pathogenesis of T2D has two main causes, insulin resistance due to β-cell dysfunction and insufficient insulin secretion [109,110]. Thus, previous studies in this field have mainly focused on β-cells. However, this approach does not address the fact that islets are composed not only of β-cells but also of endothelial cells, macrophages, and fibroblasts, among others [[111], [112], [113], [114]]. Although β-cell dysfunction is an important factor in the development of diabetes, major functional and transcriptional changes in pancreatic islet α-cells and other cells also occur in patients with diabetes [[115], [116], [117]]. These changes caused by diabetes have made it difficult to distinguish between changes in α- and β-cells alone in pancreatic tissue using previous techniques [118]. Therefore, its specific pathogenesis is not completely clear. However, in recent years, with the emergence and development of SCM-Omics and SM-Omics technologies, cell types in the pancreas can be accurately identified, and the transcriptome data of individual cells can be detected.

**Fig. 4:**
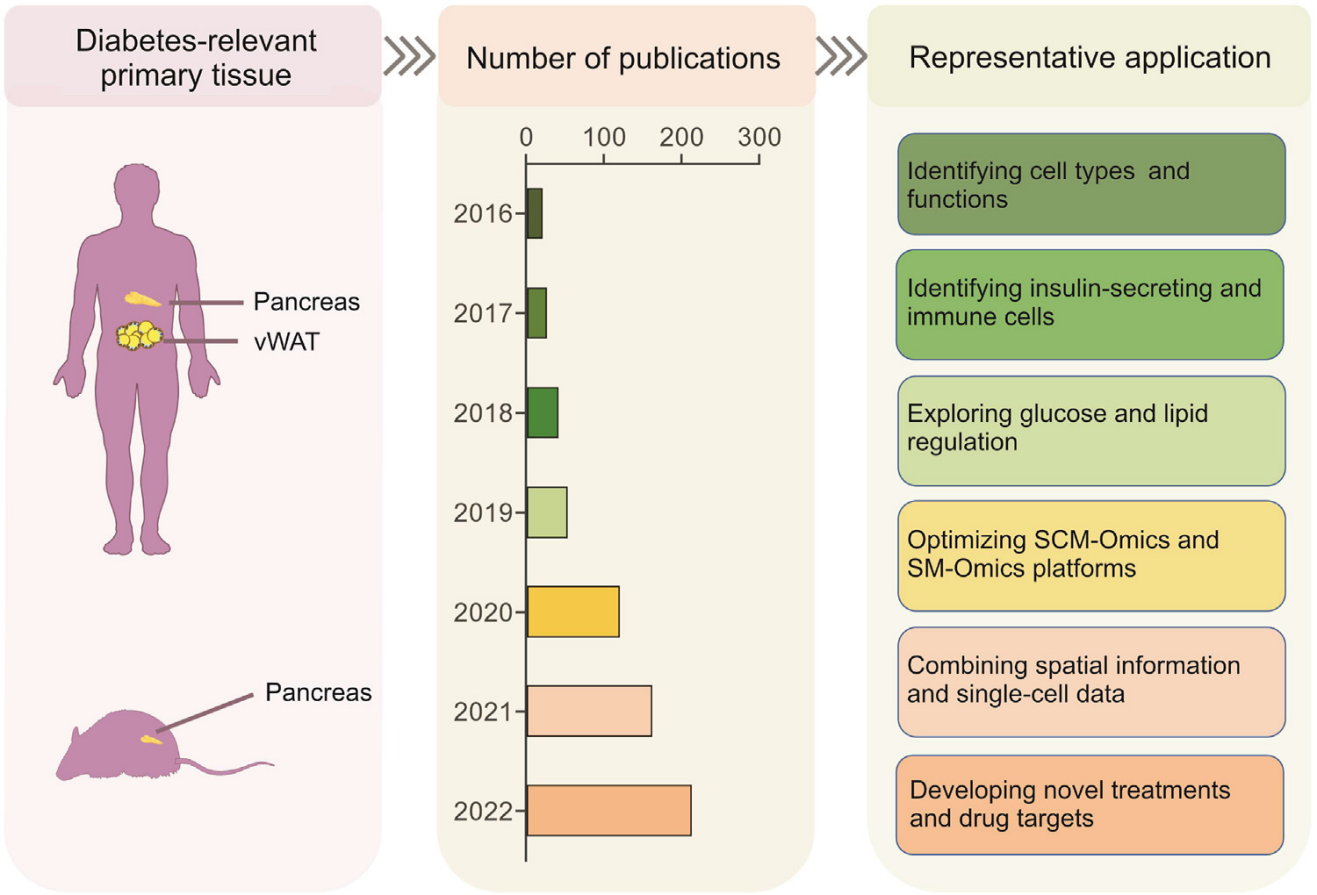
Application of single-cell multi-Omics (SCM-Omics) and spatial multi-Omics (SM-Omics) technologies in diabetes research. The application of SCM-Omics and SM-Omics technologies in primary tissues related to diabetes in humans and mice, namely the pancreas and visceral white adipose tissue (vWAT), is described. The timeline shows the number of publications on SCM-Omics and SM-Omics technologies in the field of diabetes in the PubMed database over the past seven years (2016–2022) through statistics. Representative applications in the field of diabetes research in humans and mice are described through publications related to SCM-Omics and SM-Omics technologies.

Diabetes is primarily caused by damage to the pancreas. Identifying differences in pancreatic cell types and specific genes between patients with diabetes and those without diabetes is essential for understanding how the pancreas plays a role in diabetes. The pancreas is an endocrine gland, and the islets, which consist of several types of cells, including α- and β-cells, mediate its endocrine function. Therefore, to understand the relationship between the pancreas and the occurrence of diabetes, it is crucial to mine the genetic information of each type of cell. With the emergence and development of SCM-Omics and SM-Omics, these problems will also be addressed [119]. Here, we summarize and categorize the application of SCM-Omics and SM-Omics in diabetes (Fig. 4, Table S2). Currently, the most commonly used sequencing methods in diabetes include scRNA-seq [[120], [121], [122], [123], [124], [125], [126], [127], [128], [129], [130], [131], [132], [133], [134]], snRNA-seq [[135], [136], [137], [138], [139], [140], [141], [142]], and SM-Omics sequencing [105]. The main research directions of recent studies have been identifying cell types and functions, optimizing SCM-Omics and SM-Omics platforms and algorithms, and screening specific cell groups as potential targets for the treatment of diabetes.

### 4.1 Identification of cell types and functions

SCM-Omics technologies can identify all cell types in the pancreas. By applying SCM-Omics technologies to analyze the differences between patients with diabetes and those without diabetes, cell groups or target genes that regulate diabetes can be identified. This study will help promote research on the pathogenesis of diabetes and drug development. For example, Li et al. [124] applied scRNA-seq to identify a variety of cells in the human pancreas and determined the specific expression patterns of genes in α- and β-cells. Using scRNA-seq analysis, Wang et al. [121] examined the expression patterns of major hormone genes in humans. α- and β-cells are very different, and thus, these two cell types play different roles in the pancreas. Lawlor et al. [125] used scRNA-seq to identify pancreatic endocrine and exocrine cell types in healthy individuals and T2D patients. A similar analysis by Segerstolpe et al. [123] identified subpopulations of endocrine and exocrine cell types, identified genes specific for BMI-associated cell subsets, and found that the expression of genes associated with T2D was changed in these cells. In addition, previous studies have used Western populations to provide more abundant research resources. Dorajoo et al. [120] applied scRNA-seq to detect pancreatic islet cells from three nondiabetic Singaporean Chinese individuals, advancing the understanding of the role of diabetes in the pathogenesis of East Asian individuals. These reports demonstrate that scRNA-seq technology provides new insights into islet cell type biology, facilitating the study of human islet biology and diabetes-related mechanisms.

### 4.2 Optimization of SCM-Omics and SM-Omics platforms and algorithms

SCM-Omics and SM-Omics are new technologies that have emerged in recent years, and their sequencing methods and analysis platforms are constantly being updated. It is necessary to explore the best solutions for different species or tissues and make proper adjustments. For example, researchers used the Fluidigm C1 platform to sequence pancreatic single cells in mice, and this transcriptomic approach can be used to identify novel markers and characterize rare cell subsets [130]. Muraro et al. [122] developed a new high-throughput sequencing method based on scRNA-seq technology. This method was used to establish a single-cell transcriptional map of the human pancreas and identified heat-stable antigen (HSA)/CD24 and transmembrane 4 L six family member 4 (TM4SF4) as marker genes for screening α and β cells. Basile et al. [135] compared snRNA-seq and scRNA-seq techniques and proposed that snRNA-seq is a reliable method that has the advantage of detecting the transcriptome profiles of archived frozen tissues. To explore the reasons for the reduced proliferative capacity of β cells in the postnatal period, Zeng et al. [131] measured β cell data at multiple postnatal time points by scRNA-seq and obtained a transcriptomic map of islet developmental trajectories and β cell state changes. Based on this, Sharon et al. [132] used a new algorithm to construct a trajectory to describe the transcriptional changes that occur during islet formation in endocrine progenitors. In addition, Duvall et al. [142] performed single-cell transcriptomics analysis, chromatin accessibility analysis combined with gene tagging and other technical analyses to identify the transcription factor Neurog3 as a pioneer transcription factor of the pancreatic endocrine lineage. This approach lays the foundation for the subsequent joint analysis of multiple techniques to identify the pathogenesis of the disease. Bäckdahl et al. [105] applied spatial transcriptome technology to not only parse the spatial transcriptional map of human adipose tissue but also discover a subpopulation of adipocytes associated with insulin sensitivity. These findings suggest that targeting this cell population could improve insulin resistance in adipose tissue. Therefore, developing new technologies and analytical methods is crucial to improving our understanding of the occurrence of diabetes.

### 4.3 Identification of specific cell groups or genes as potential targets for the treatment of diabetes

Identifying specific cell groups or target genes as potential targets for the treatment of diabetes has become the latest research strategy in this field. Specific cell groups have been identified using scRNA-seq technology, and their specifically expressed genes were then screened as potential targets for the treatment of diabetes. For example, Wang et al. [137] applied scRNA-seq to identify a new type of endocrine progenitor cell in the mouse pancreas; thus, the potential roles of such cells in the treatment of diabetes can be explored. The islet single-cell map of T1D was established using scRNA-seq by Zakharov et al. [140]. This map was reanalyzed by Sona et al. [143] to identify the specific, high expression of Cadm1 in the islet myeloid cell population during T1D development, and then, genetic and pharmacological approaches were used to explore the mechanisms underlying the role of Cadm1 in T1D development. Fukaishi et al. [139] analyzed pancreatic islets using a highly sensitive method, droplet-assisted RNA targeting by single-cell sequencing (DART-seq), to identify a novel glucagon-like peptide-1 (GLP-1)-mediated pathway in humans that regulates α-cell function. Therefore, the identification of these unique cell subsets and target genes, as the object of study of islet function, will introduce new strategies for treating diabetes.

In conclusion, with the development of SCM-Omics and SM-Omics technologies in recent years, their application in diabetes research has received increasing attention. These two technologies provide researchers with a more comprehensive and in-depth understanding of the mechanism of diabetes occurrence and development. First, SCM-Omics technology can help scientists better understand the changes in the expression profile of different cell types during the occurrence of diabetes. Second, SM-Omics technology also plays an essential role in diabetes research. However, the application of these two technologies still faces some challenges, such as complex preprocessing and data analysis, potential biases and errors in existing analysis methods, and the multifactorial and complex nature of diabetes. In general, the application of SCM-Omics and SM-Omics technologies in diabetes research is significant, but further optimization and promotion are needed for more effective support for treating and preventing diabetes.

## 5 The application of SCM-Omics and SM-Omics in NAFLD

NAFLD has become the most prevalent liver disease worldwide, affecting more than 25% of the global population [[144], [145], [146]]. NAFLD can progress to hepatitis, liver fibrosis, and even liver cancer [147,148]. With the improvement of living standards, the incidence of NAFLD is also rising, which seriously affects human life and health [149]. Although numerous studies have shown that NAFLD is closely related to obesity and its related metabolic diseases [150,151], some factors (genetics, nutrition, environment, etc.) leading to NAFLD have also been identified [152,153]. However, its pathogenesis is largely incompletely understood. Currently, there are no drugs that can treat NAFLD, and patients urgently need such treatments [154]. Therefore, finding effective research methods to analyze the pathogenesis of NAFLD is the focus of current research. Normally, the occurrence of NAFLD is accompanied by the occurrence of liver tissue fibrosis. To understand the fibrosis process more broadly and to decipher the molecular mechanisms that accompany fatty liver-related fibrosis, many transcriptomic studies have been conducted in animal models and humans [155,156]. Although many genes and regulatory pathways associated with liver fibrosis and inflammation have been identified, these liver tissue transcriptomic studies were unable to distinguish between those types of cells that are responsible for all common fibrotic changes and inflammation. Therefore, exploring the compositional changes and differential gene expression patterns in hepatic cells under pathological conditions is essential to reveal the pathogenesis of fatty liver and to provide new therapeutic approaches. The application of SCM-Omics and SM-Omics technologies in recent years has enabled us to further understand the human liver in an unprecedented way, thus providing more research ideas and therapeutic approaches for the treatment of NAFLD.

**Fig. 5:**
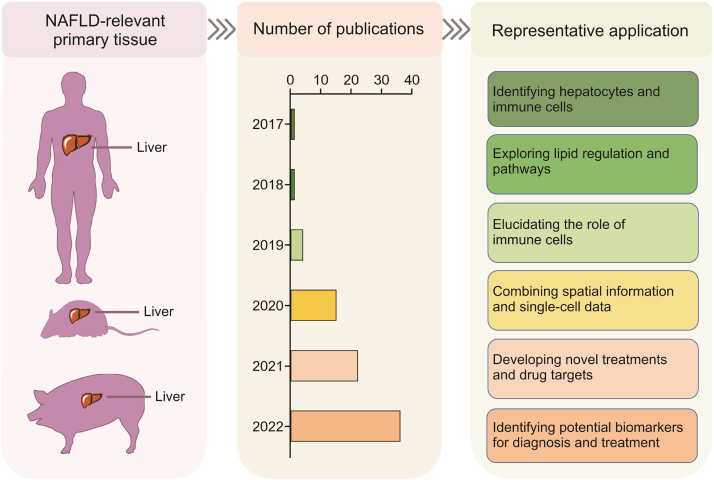
Application of single-cell multi-Omics (SCM-Omics) and spatial multi-Omics (SM-Omics) technologies in non-alcoholic fatty liver disease (NAFLD) research. The application of SCM-Omics and SM-Omics technologies in NAFLD-associated primary tissue, namely the liver, in humans, mice, and pigs. The timeline shows the number of publications on SCM-Omics and SM-Omics techniques in the field of NAFLD in the PubMed database over the past six years (2017–2022). Representative applications in the field of NAFLD research in humans, mice, and pigs are described through publications related to the SCM-Omics and SM-Omics technologies.

SCM-Omics and SM-Omics techniques have been applied to reveal differences in cell populations in the livers of NAFLD patients and healthy individuals, and a series of tools have been developed to obtain genetic information on cell types and spatial locations [12]. Combining new technologies, such as spatial transcriptomics, will further deepen our understanding of the pathogenesis of NAFLD. The application of scRNA-seq and SM-Omics in humans [[157], [158], [159], [160], [161]], mice [[162], [163], [164], [165], [166], [167], [168]], and minipigs [169] has yielded many valuable results in the field of NAFLD research (Fig. 5). For example, Wang et al. [157] performed single-cell assays of healthy human liver samples to reveal major regulators of cell-cell interactions and genes potentially related to hepatic fibrosis. Using scRNA-seq, Su et al. [162] found that Kupffer cells have distinct expression profiles from monocyte-derived macrophages during the development of NAFLD. The data suggest that Kupffer cells have a stronger immune role, whereas the derived macrophages have a strong ability to differentiate. These findings advance our understanding of cellular heterogeneity in NAFLD. Park et al. [168] applied droplet-sequencing (Drop-seq) to describe the single-cell transcriptional profiling of livers with high-fat diet-induced NAFLD. These latest studies provide a useful resource for understanding hepatocyte alterations at the single-cell level in NAFLD.

Here, we summarize and categorize the studies applying SCM-Omics and SM-Omics techniques in NAFLD (Table S3). At present, the main sequencing methods used in NAFLD research include scRNA-seq [[157], [158], [159]], snRNA-seq [[159], [160], [161]], and spatial transcriptome sequencing [159,162]. SCM-Omics and SM-Omics technologies have provided new research directions for truly understanding the heterogeneity and complexity of NAFLD disease progression, but the application of multi-omics analysis remains in the stage of continuous renewal and development. For example, the major functions of the liver are regulated by the interaction of hepatocytes and nonparenchymal cells. However, current techniques for cell isolation and on-machine sequencing limit our ability to capture all the cells in the liver, thereby limiting our ability to comprehensively analyze the liver. To address this issue, Andrews et al. [159] analyzed human liver tissue by coapplying scRNA-seq and snRNA-seq techniques. The differences in the analysis results obtained with the two techniques were compared, and new cell subsets were discovered. The spatial distribution of these cells was then verified by separate spatial transcriptome sequencing and immunohistochemistry. The application of these multi-omics technologies not only provides a new method for analyzing the SCM-Omics map of the livers of humans and animal models but also provides a new method for understanding the pathogenesis of NAFLD and screening new therapeutic targets.

SCM-Omics and SM-Omics technologies open new avenues for truly understanding the molecular mechanisms involved in NAFLD pathogenesis. In the future, combined multi-omics analyses could yield more comprehensive and accurate data, as demonstrated by Diamanti et al. [160]. The integration of snRNA-seq data led to the discovery of a small cluster of inactive hepatic stellate cells, suggesting that snRNA-seq can be used in this analysis. Single-cell sequencing should be used to explore spatial and temporal factors, which will lead to important breakthroughs in the future. The application of scRNA-seq data from public databases is also an important research direction in the future and can be analyzed according to specific cell populations. For example, Zhang et al. [169] increased the credibility of such research results using the data from two groups of different species and simultaneously analyzed the public database to improve the data utilization of public databases. This approach can also be applied to the study of single genes. Gwag et al. [167] reanalyzed the public scRNA-seq datasets of mouse liver and human liver tissues and found that TSP1 is highly expressed in a special cell subset. This finding lays the foundation for follow-up research directions. In addition, NAFLD disease mechanisms can be more precisely studied by combining spatial information, as demonstrated by Halpern et al. [164] Combining scRNA-seq technology with in situ detection of the expression of landmark genes was applied to study mouse livers, and the spatial division of labor in the liver has been reconstructed and revealed in a mammalian model. Andrews et al. [159] performed a systematic comparative analysis using a combination of SCM-Omics and SM-Omics to provide a single-cell map of the liver of healthy humans. Combining molecular biology validation methods and drug screening is important for providing transformable drugs for the targets obtained by SCM-Omics and SM-Omics. In conclusion, these new research directions provide more potential targets and more precise therapeutic approaches for the treatment of fatty liver.

In conclusion, SCM-Omics and SM-Omics technologies have been widely applied in NAFLD research, providing new approaches to problems that traditional methods cannot solve. SCM-Omics enables comprehensive sequencing analysis of all cells, identifying changes in transcription factors and regulatory networks between different subgroups of cells, revealing unique advantages for understanding NAFLD pathogenesis. Through single-cell transcriptome sequencing, researchers have identified specific subgroups of cells in the NAFLD liver that display coordinated changes in transcription factors, helping reveal the molecular mechanism of NAFLD pathogenesis. SM-Omics simultaneously determine the gene expression and spatial location of cells, offering new approaches to finding new targets for treatment. Through spatial sequencing analysis, researchers can determine the 3D spatial distribution of liver cells and other cell types, providing information on the spatiotemporal distribution of liver cell changes. However, challenges remain, including technical limitations and the need for improved data processing and modeling techniques. Overall, these technologies provide an essential molecular biological basis for understanding NAFLD pathogenesis, with future potential for critical roles in treatment and studies. Further improvements are needed to promote their wider application, including the development of predictive models and the improvement of diagnostic and therapeutic accuracy. In conclusion, SCM-Omics and SM-Omics technologies offer significant potential to advance our understanding of NAFLD pathogenesis, paving the way for improved diagnosis and treatment options.

## 6 The application of SCM-Omics and SM-Omics in CVD

**Fig. 6:**
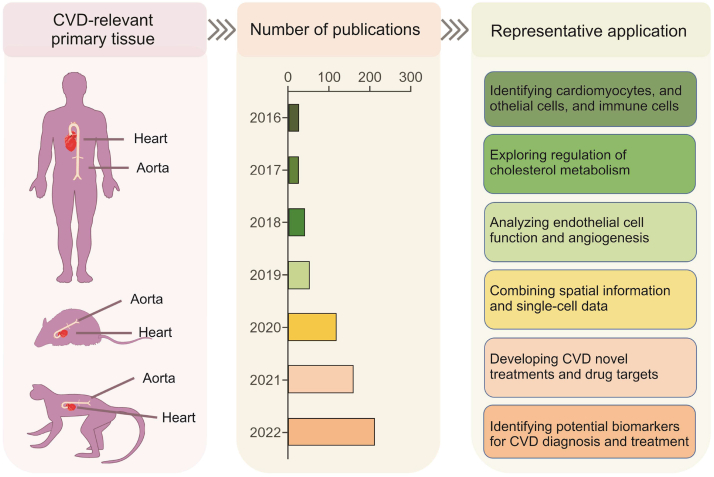
Application of single-cell multi-Omics (SCM-Omics) and spatial multi-Omics (SM-Omics) technologies in cardiovascular disease (CVD) research. The application of SCM-Omics and SM-Omics technologies to CVD-relevant primary tissues, namely the heart and aorta, in humans, mice, and pigs. The timeline shows the number of publications on SCM-Omics and SM-Omics techniques in the field of CVD research in the PubMed database over the past seven years (2016–2022). Representative applications in the field of CVD research in humans, mice, and pigs are described through publications related to the SCM-Omics and SM-Omics technologies.

CVD has the highest mortality rate globally, with an estimated 17.9 million CVD-related deaths occurring every year [170]. Atherosclerosis is the main cause of CVD pathogenesis [171], and non-high-density lipoprotein cholesterol (non-HDL-c) is a predictor of CVD risk [172,173]. Therefore, reducing the level of non-HDL-c in the blood is an effective means to prevent and treat atherosclerosis. Although the level of non-HDL-c in the blood is affected by many factors, such as nutritional, environmental, and genetic factors, 50% of its phenotypic variation is determined by genetic factors [174]. It has been found that low-density lipoprotein receptor (LDLR) [175,176], asialoglycoprotein receptor 1 (ASGR1) [177,178], proprotein convertase subtilisin kexin 9 (PCSK9) [179,180], LIM domain and actin binding 1 (LIMA1) [181], the cholesterol transporter Niemann-Pick C1-like protein 1 (NPC1L1) [182,183] and other key genes are involved in the occurrence of atherosclerosis. However, the genetic regulation of atherosclerosis is complex and needs further exploration. With the rapid development of SCM-Omics and SM-Omics technologies, the progress of CVD research has dramatically improved. In recent years, SCM-Omics technology has been widely used in CVD research (Fig. 6). For example, in myocardial infarction, SCM-Omics technology can reveal the cell heterogeneity in the myocardial infarction area, such as obtaining the RNA sequence of myocardial injury cells will help researchers further understand the molecular mechanism of myocardial injury. Similarly, vascular endothelial cells are ubiquitous in CVD, and SCM-Omics technology makes it easier to analyze vascular endothelial cell differentiation and function. The development of SM-Omics technology has also promoted progress in CVD research. In the past, researchers only labeled and analyzed the surface layer of cardiovascular tissue, and could not obtain information about individual cells inside the tissue. However, the current SM-Omics technology makes it feasible to analyze cells of different cell types and in different regions, thereby promoting the understanding of CVD pathology and the discovery of therapeutic methods. The application of these two techniques has allowed researchers to obtain more detailed and comprehensive information, which can help unravel some of the mysteries of the complexity of CVD and find new treatments. As the technology continues to be improved and promoted, these two technologies will play a more critical role in future CVD research.

In recent years, SCM-Omics technology has provided new insights into CVD. SCM-Omics technology in combination with SM-Omics technology has been applied to identify cell types in the heart and blood vessels and to construct cellular spatial omics profiles of the heart and blood vessels in humans and animal models [[184], [185], [186], [187], [188]]. Many breakthroughs have been made toward elucidating heart and vascular-related diseases. Here, we summarize the latest findings in CVD obtained using SCM-Omics and SM-Omics technologies (Table S4). At present, the main sequencing methods used in CVD research include scRNA-seq [186,187,[189], [190], [191], [192], [193], [194], [195], [196], [197], [198], [199], [200], [201], [202], [203], [204], [205], [206]], snRNA-seq [188,[207], [208], [209]], and spatial transcriptome sequencing [184,185,210,211]. Additionally, the advantages of combined multi-omics analyses and the prospect of applying this method to the precision treatment of CVD and the development of appropriate drugs are reviewed.

SM-Omics and SCM-Omics technologies are widely used in the study of heart disease to construct cardiac cell atlases from human and animal models, providing a solid foundation for understanding cardiac development and treating cardiac diseases. The current major discovery is the identification and classification of cardiac cells. Tucker et al. [184] performed an snRNA-seq analysis to examine the human heart, and identified nine major cellular taxa and 20 cellular subclasses in the heart. By combining scRNA-seq and snRNA-seq with in situ imaging techniques and machine learning, Litviňuková et al. [185] provided detailed insights into the cardiomyocyte library and highlighted specific compartmental characteristics and differences between male and female donors. In addition, Asp et al. [188] used a single-cell spatiotemporal approach to study human heart development for the first time and developed a new method to explore the developmental processes of various tissues and organs. These results clearly demonstrate that the integration of spatial and temporal information with single-cell gene expression data is critical for identifying key differences between cell types and for the detailed analysis of developing tissues. Additionally, Kupe et al. [208] applied SCM-Omics technology to jointly analyze cardiac tissue and characterize cell type-specific changes in gene regulation, providing a comprehensive SM-Omics atlas of the heart. Recent research in mouse models has also led to breakthroughs. Farbehi et al. [196] performed scRNA-seq of the mouse heart and identified more than 30 cell subtypes, including previously unknown taxa. In this study, a group of fibroblasts was found to highly express the Wif1 gene. Previous studies of the Wif1 gene have shown that it plays an important regulatory role in cardiac injury. Further experiments showed that this group of cells can control the timing of cardiac repair after injury. These studies demonstrate the significant clinical application of these methods. In addition to new advances in the non-human primate (NHP) *Macaca fascicularis*, Han et al. [205] obtained a large-scale single-cell and mononuclear transcriptome map containing over 1 million cells from 45 tissues of the NHP *Macaca fascicularis* that included cardiovascular-related tissues (heart, aorta, and carotid arteries). This dataset provides a large, carefully annotated resource for studying species phylogenetically close to humans. These studies lay the groundwork for an understanding of human heart development and disease and offer potential clues for the treatment of heart-related diseases.

SCM-Omics and SM-Omics technologies are also widely used in the study of vascular diseases. Usually, atherosclerosis is the main cause of CVD. However, atherosclerosis is considered to be an inflammatory disease involving interactions between immune cells, vascular endothelial cells, and smooth muscle cells [212]. SCM-Omics and SM-Omics can accurately detect the genetic information of single cells and have been used to detect the heterogeneity of various cell populations, including macrophages, in atherosclerotic tissues of mouse models [194,199] and carotid atherosclerotic tissues of humans [191]. Numerous studies have revealed a large-scale single-cell and mononuclear transcriptome map containing over 1 million cells from multiple tissues of humans, the NHP *Macaca fascicularis*, and mice [195,197,205]. These datasets constitute a comprehensive cellular atlas of blood vessels and vessel-related cell types in model animals, and thereby provide a solid foundation for future studies of vascular development and disease.

Before the advent of single-cell technology, numerous studies demonstrated the leukocyte functions involved in atherosclerosis. In atherosclerosis, leukocytes play an essential role in the composition and function of the vascular system. Macrophages are the most abundant leukocytes in any lesion and are the most significant factor affecting the size of the lesion [213]. Winkels et al. [193] found that aortic leukocytes have a similar complexity to leukocytes in lymphoid organs and identified several novel leukocyte subsets that are differentially regulated in disease and may predict CVD in humans. Cochain et al. [194] applied scRNA-seq technology to reveal the transcriptional profile and heterogeneity of cells in atherosclerosis of the aorta and identified novel macrophage populations. Lin et al. [200] demonstrated a range of macrophage activation states using scRNA-seq, and the complexity of macrophages was reported to be far beyond the previously proposed sputum (M1) and BAL (M2)-derived macrophages polarization states [214]. These studies also identified a new class of monocytes and showed that monocytes in inflamed tissues are not differentiated into macrophages but are in a constant state of renewal. With the development of technology, three main macrophage clusters were gradually identified, and these clusters included resident macrophages, proinflammatory macrophages, and foamy high expression of triggering receptor expressed on myeloid cells 2 (TREM2 ^hi^) macrophages [194,201,215]. Later, McArdle et al. [209] combined quantitative in vivo imaging and scRNA-seq to identify four vascular macrophage subpopulations with distinct transcriptomic profiles in atherosclerosis that did not overlap with macrophage subpopulations in other studies Four major macrophage subsets have been identified thus far, and from these studies, both murine and human plaques appear to contain proinflammatory, foamy anti-inflammatory, and resident macrophages. Therefore, future studies using mouse models are needed to dissect the role of the different macrophage subsets in the development of atherosclerosis.

The causes of vascular disease have been investigated at the single-cell level. Several studies have demonstrated the distribution of cellular taxa in vascular diseases and their relevance to diseases using SCM-Omics technologies. Such studies have also identified abnormal cell subsets in vascular disease, assessed vascular disease-specific regulatory networks, and identified vascular disease-specific regulatory networks. The related key genes were identified as potential therapeutic agents for vascular diseases by network pharmacology [191,203,216]. SCM-Omics and SM-Omics technologies have enhanced our understanding of cell types and the spatial location of gene expression in tissues and organs. Atherosclerotic plaques contain multiple cell types. Zhang et al. [192] applied scRNA-seq technology to assess atherosclerotic plaques in human carotid arteries and identified specific macrophage-like cells. In addition, Zhao et al. [202] reported a novel scRNA-seq method to probe the heterogeneity of cardiovascular endothelial cells in a mouse model of atherosclerosis. These studies improve our understanding of the disease processes and mechanisms and further advance drug development for the treatment of arterial atherosclerosis.

In conclusion, SCM-Omics and SM-Omics technologies are essential for studying CVD, providing new methods for treatment and prevention by analyzing gene expression, protein composition, cellular status, and spatial location. SCM-Omics technology enables in-depth exploration of cell types, molecular mechanisms, and disease processes, while single-cell protein analysis provides accurate predictive models for early diagnosis and treatment. SM-Omics technology can correlate biomolecule expression with spatial location to deepen the understanding of CVD structure and pathogenesis, and study the interaction between the microenvironment of the heart and cardiomyocytes. However, these technologies still face challenges and issues, such as sample processing and data accuracy, requiring long-term exploration and accumulation. Improvements in data analysis and image processing of biological samples are also necessary. Despite these challenges, these technologies have achieved significant results in CVD research and will continue to make meaningful contributions to cardiovascular health with continuous improvement and development.

## 7 Conclusion and future outlook

SCM-Omics and SM-Omics techniques are developing rapidly and are widely used in various fields in the life sciences. Additionally, as computational tools and SCM-Omics and SM-Omics technologies continue to emerge, multi-omics data are becoming increasingly integrated into bioinformatics analyses, providing us with more detailed information. These techniques provide further insight into the molecular pathways involved in metabolic diseases, such as obesity, diabetes, fatty liver, and CVD, and facilitate rapid screening of therapeutic targets. In recent years, several spatial transcriptomic approaches have been developed. The disadvantages of scRNA-seq, such as loss of histological information, have been overcome primarily by barcoded arrays of microdissected tissue with transcript capture and in situ sequencing. The combination of SCM-Omics and SM-Omics techniques will also improve our understanding of diseases by spatially and temporally uncovering the transcription of single cells in complex tissues and organs. Furthermore, with the advent of the era of precision medical treatment, SCM-Omics and SM-Omics technologies will soon become powerful tools for routine clinical diagnosis and personalized medicine. SCM-Omics techniques can help researchers understand different cell subtypes and their biological roles in certain diseases. Through the use of SCM-Omics techniques, it is possible to understand the complex interactions between different cells and their subtypes as well as the role of these cells in an individual's immune response. The use of SM-Omics technology can help reveal the pathological changes and intercellular relationships of certain diseases, which would improve the diagnosis and treatment of diseases. These technologies have been clinically applied to diagnose and treat conditions such as lung and breast cancer. In the future, SCM-Omics and SM-Omics technology will be further used for the personalized diagnosis and treatment of metabolic diseases to improve diagnostic accuracy and therapeutic effects.

However, some problems and challenges exist in the application of SCM-Omics and SM-Omics technologies. First, data processing and analysis complexity are important challenges of SCM-Omics technologies. Since the sample size of single cells is usually large, it needs to be processed using high-throughput techniques. However, the existing data analysis tools cannot handle such large-scale data effectively. Reproducibility, standardization, and quality control of single-cell data are also challenging. Second, although SM-Omics techniques can accurately describe the distribution and spatial location of different cell types in tissues, the current technologies still cannot accurately detect and identify some cell types and molecular distributions, which may affect their use in clinical diagnosis. Therefore, further technical improvements and optimizations are needed. In addition, the application of SCM-Omics and SM-Omics technology faces the problem of high implementation costs, which include purchasing, maintaining, and operating equipment and spending on associated analytics and algorithms. Furthermore, the time and cost of performing single-cell analysis on many patients are also issues to consider when applying this technology. Personal privacy and data protection are also issues that need attention. Since SCM-Omics and SM-Omics technologies involve private personal data such as genomes and biomarkers, more stringent and reliable protection measures are needed to protect the privacy and rights of patients and participants.

In conclusion, SCM-Omics and SM-Omics technologies will be important trends and development directions in the medical field in the future. These methods have extensive application prospects and can improve the effectiveness and accuracy of routine clinical diagnosis and personalized medicine. However, problems and challenges regarding technical processing, data quality, cost, and privacy protection still need to be resolved. In response to such problems, all parties need to work together to find more reliable and sustainable solutions to promote the application of these technologies in routine clinical diagnosis and personalized medicine for metabolic diseases.

## CRediT author statement

**Baocai Xie:** Investigation, Writing - Original draft preparation, Reviewing and Editing, Visualization, Funding acquisition; **Dengfeng Gao:** Writing - Original draft preparation, Data curation; **Biqiang Zhou:** Data curation, Visualization; **Shi Chen:** Supervision, Writing - Reviewing and Editing, Funding acquisition; **Lianrong Wang:** Supervision, Writing - Reviewing and Editing, Funding acquisition.

## Declaration of competing interest

The authors declare that there are no conﬂicts of interest.
