# Supplementary material for: New discoveries in the field of metabolism by applying single-cell and spatial omics
Source: J Pharm Anal. 2023 Jun 4;13(7):711–25. doi: 10.1016/j.jpha.2023.06.002 (PMC10422156; doi:10.1016/j.jpha.2023.06.002)
Supplement: Multimedia component 1 [file mmc1.docx]

**Supplementary Information**

**New discoveries in the field of metabolism by applying Single-cell and Spatial Omics**

Baocai Xie, Dengfeng Gao, Biqiang Zhou, Shi Chen, Lianrong Wang

Correspondence to: [lianrong@whu.edu.cn](mailto:lianrong@whu.edu.cn) (L, Wang)

[shichen@email.szu.edu.cn](mailto:shichen@email.szu.edu.cn) (S, Chen)

**Table of contents**

[Supplementary Table 1. Inductive SCM-Omics and SM-Omics technologies in obesity. 2](#_Toc137579645)

[Supplementary Table 2. Summary of the application of SCM-Omics and SM-Omics technologies in diabetes. 5](#_Toc137579646)

[Supplementary Table 3. Application of inductive SCM-Omics and SM-Omics technologies in NAFLD. 9](#_Toc137579647)

[Supplementary Table 4. Summary of the application of SCM-Omics and SM-Omics technologies in CVD. 12](#_Toc137579648)

[Supplementary References 16](#_Toc137579649)

# Supplementary Table 1. Inductive SCM-Omics and SM-Omics technologies in obesity.

| Species | Sequencing method | Tissues | Platform | Samples | Number of captured cells / Tissue-covered spot | Main findings | Ref. |
| --- | --- | --- | --- | --- | --- | --- | --- |
| Human | scRNA-seq | sWAT | 10× Genomics | 1 | 11338 | Human adipose tissue contains analogous stromal populations with mice | [1] |
|  |  | WAT | 10× Genomics | 6 | 82577 | Provide a detailed and unbiased cellular landscape of homeostatic and inflammatory circuits in healthy human WAT | [2] |
|  |  | vWAT | 10× Genomics | 12 | 26350 | Provide a rich catalog of cell types residing in adipose tissue including both latent and common cell populations. | [3] |
|  |  | sWAT |  | 13 |  |  |  |
|  |  | sWAT | Fluidigm C1 system | 6 | 574 | Map adipose tissue stem cell populations in human subcutaneous WAT | [4] |
|  |  | sWAT | 10× Genomics | 9 | 28465 | BMI was negatively correlated with the relative abundance of adipocytes and positively correlated with ASPCs and myeloid cells | [5] |
|  | snRNA-seq | sWAT/ vWAT |  | 13 | 137684 |  |  |
|  |  | BAT | 10× Genomics | 16 | 36590 | Reveals a subpopulation of adipocytes that regulates thermogenesis | [6] |
|  |  | sWAT |  | 5 | 2438 |  |  |
|  | Full-length sc / snRNA-seq | sWAT | iCELL8 | 2 | 2253 | Provide critical evidence for the utility of sc full-length transcriptomics in WAT and SVF in humans. | [7] |
|  | ST | sWAT | 10× Genomics | 10 | 27937 | Spatial mapping reveals human adipocyte subpopulations with distinct sensitivities to insulin | [8] |
| Mouse | scRNA-seq | sWAT | 10× Genomics | 6 | 11423 | DPP4+ cells are multipotent mesenchymal progenitors | [1] |
|  |  | eWAT |  | 5 | 1045 | Unraveling the cellular and molecular determinants of WAT | [9] |
|  |  | Periaortic adipose tissue |  | 5 | 12158 | Reveal the Contribution of Perivascular Adipose Tissue Stem Cells to Vascular Remodeling | [10] |
|  |  | iWAT |  | 4 | 16023 | Identify cell types and map adipogenic trajectories | [11] |
|  |  | eWAT |  | 4 | 17640 |  |  |
|  |  | eWAT |  | 4 | 4779 | APCs are major contributors to obesity-associated increases in adipose tissue mass | [12] |
|  |  | sWAT/ vWAT |  | 6 | 1084 | CD142+ cells are adipogenesis-regulatory cells | [13] |
|  |  |  | Fluidigm C1 system | 3 | 208 |  |  |
|  | snRNA-seq | iWAT | 10× Genomics | 3 | 54000 | Identified a thermogenic adipocyte subtype that was enriched in IL10Rα-deficient mice. | [14] |
|  |  | iWAT |  | 4 | 91252 | Provide an initial blueprint for a comprehensive set of interactions between individual cell types in the adipose niche in leanness and obesity. | [5] |
|  |  | eWAT |  | 4 | 106469 |  |  |
|  |  | BAT/ iWAT/ vWAT |  | 7 | 28771 | Reveals a subpopulation of adipocytes that regulates thermogenesis | [6] |
|  |  | eWAT |  | 3 | 12000 | Uncovers All Major Cell Types in Epididymal Adipose Tissue | [15] |

scRNA-seq: single-cell RNA sequencing; snRNA-seq: single-nucleus RNA sequencing; ST: spatial transcriptomics; sWAT: subcutaneous white adipose tissue; WAT: white adipose tissue; vWAT: visceral white adipose tissue; BAT: brown adipose tissue; eWAT: epididymal white adipose tissue; iWAT: inguinal white adipose tissue; Ref.: references; BMI: body mass index; ASPCs: abundance of adipose stem and progenitor cells; DPP4^+^: dipeptidyl peptidase 4 positive; APCs: antigen-presenting cells; CD142^+^: tissue factor positive.

# Supplementary Table 2. Summary of the application of SCM-Omics and SM-Omics technologies in diabetes.

| Species | Sequencing method | Tissues | Platform | Samples | Number of captured cells / Tissue-covered spot | Main findings | Ref. |
| --- | --- | --- | --- | --- | --- | --- | --- |
| Human | scRNA-seq | Islets | Fluidigm C1 | 3 | 448 | Identify expression signatures in East-Asian β-cells that perhaps | [16] |
|  |  | Islets | Fluidigm C1 | 8 | 635 | Single-Cell Transcriptomics of the Human Endocrine Pancreas | [17] |
|  |  | Pancreas | SORT-seq | 4 | 4,262 | A Single-Cell Transcriptome Atlas of the Human Pancreas | [18] |
|  |  | Islets | Smart-seq2 | 10 | 2,209 | Single-Cell Transcriptome Profiling of Human Pancreatic Islets in Health and Type 2 Diabetes | [19] |
|  |  | Islets | Smart‐seq2 | 1 | 70 | Single‐cell transcriptomes reveal characteristic features of human pancreatic islet cell types | [20] |
|  |  | Islets | Fluidigm C1 | 8 | 638 | Single-cell transcriptomes identify human islet cell signatures and reveal cell-type-specific expression changes in type 2 diabetes | [21] |
|  |  | Pancreatic cells | 10× Genomics | 1 | 2,999 | SLC26A9 is predominantly expressed in pancreatic ductal cells and frequently coexpressed with CF transmembrane conductance regulator (CFTR) along with transcription factors that have binding sites 5′ of SLC26A9 | [22] |
|  |  | Blood | HiSeq2500 | 4 | 2,767 | Autoreactive T cell receptors with shared germline-like α chains in type 1 diabetes | [23] |
|  |  | Islets | DART-Seq | 3 |  | Validate a high-sensitivity method for scRNA-seq in human islets and identify a potentially novel GLP-1-mediated pathway regulating human α cell function. | [24] |
|  |  | Islets | DROP-Seq | 3 | – |  |  |
|  |  | Pancreatic cells | 10× Genomics | 3 | 5,288 | 3D chromatin maps of the human pancreas reveal lineage-specific regulatory architecture of T2D risk | [25] |
|  | snRNA-seq | Islets | 10× Genomics | 1 | 2253 | Using single-nucleus RNA-sequencing to interrogate transcriptomic profiles of archived human pancreatic islets | [26] |
|  | ST | WAT | 10× Genomics | 10 | 27,937 | Spatial mapping reveals human adipocyte subpopulations with distinct sensitivities to insulin | [8] |
| Mouse | scRNA-seq | Pancreatic cells | Fluidigm C1 | 1 | 96 | Single cell transcriptomic profiling of mouse pancreatic progenitors | [27] |
|  |  | Pancreatic β-cells | Fluidigm C1 | 47 | 387 | First high-resolution molecular characterization of state changes in postnatal beta-cells and paves the way for the identification of novel therapeutic targets to stimulate beta-cell regeneration. | [28] |
|  |  | Islet precursors | Smart-seq | 1 | 440 | Single-cell spatiotemporal studies of the developing pancreas reveal a tight link between morphology and endocrine cell differentiation, with α and β cells forming layered peninsular structures. | [29] |
|  |  | Pancreas | 10× Genomics | 4 | 6,813 | Single-cell transcriptome analysis defines heterogeneity of the murine pancreatic ductal tree | [30] |
|  |  | Islet cells | Fluidigm C1 | 1 | 622 | Provide a resource for identification of high-quality gene expression datasets to help expand insights into genes and pathways characterizing islet cell types. | [31] |
|  |  | Pancreatic cells | 10× Genomics | 1 | 6,626 | Single-Cell Transcriptome Profiling of Mouse and hESC-Derived Pancreatic Progenitors | [32] |
|  | snRNA-seq | Islets | 10× Genomics | 3 | 7,160 | Describe a previously unidentified protein C receptor positive (Procr+) cell population in adult mouse pancreas | [33] |
|  |  | Islets | 10× Genomics | 6 | 12,430 | Targeted pharmacological therapy restores β-cell function for diabetes remission | [34] |
|  |  | Islets | 10× Genomics | 4 | 3,949 | Characterisation of Ppy-lineage cells clarifies the functional heterogeneity of pancreatic beta cells in mice | [35] |
|  |  | Pancreatic islets | 10× Genomics | 9 | 42,140 | Single-cell RNA sequencing of murine islets shows high cellular complexity at all stages of autoimmune diabetes | [36] |
|  |  | Islet | 10× Genomics | 5 | 19,640 | Single-Cell Landscape of Mouse Islet Allograft and Syngeneic Graft | [37] |
|  |  | Islet | Smart‐seq2 | 3 | 604 | Single-cell transcriptome and accessible chromatin dynamics during endocrine pancreas development | [38] |

–: no data; scRNA-seq: single-cell RNA sequencing; snRNA-seq: single-nucleus RNA sequencing; ST: spatial transcriptomics; SORT-seq: single-cell orthogonal RNA sequencing; DART-seq: droplet-assisted RNA targeting by single-cell sequencing; DROP-seq: droplet sequencing; WAT: white adipose tissue; Ref.: references; SLC26A9: solute carrier family 26 member 9; GLP-1: glucagon-like peptide-1; 3D: three-dimensional; T2D: type 2 diabetes.

# Supplementary Table 3. Application of inductive SCM-Omics and SM-Omics technologies in NAFLD.

| Species | Sequencing method | Tissues | Platform | Samples | Number of captured cells / reads | Main findings | Ref. |
| --- | --- | --- | --- | --- | --- | --- | --- |
| Human | scRNA-seq | Liver | 10× Genomics | 6 | 17,810 | This study highlights potential cell–cell interactions and master regulators that underlie HSC activation and reveals genes that may represent prospective hallmark signatures for liver fibrosis. | [39] |
|  |  | Liver | 10× Genomics | 5 | 8,444 | Identified 20 distinct cell clusters including two distinct populations of liver‐resident macrophages with immunoregulatory and inflammatory properties. | [40] |
|  |  | Liver | 10× Genomics |  | 22,374 | ScRNA-seq reveals maladaptation of human cirrhotic vascular endothelial cells | [41] |
|  |  | Liver | 10× Genomics | 4 | 29,432 | Single‐Cell, Single‐Nucleus, and Spatial RNA Sequencing of the Human Liver Identifies Cholangiocyte and Mesenchymal Heterogeneity | [42] |
|  | snRNA-seq | Liver | 10× Genomics | 4 | 43,863 |  |  |
|  | ST | Liver | NovaSeq 6000 | 4 | 167,400,637 |  |  |
|  | snRNA-seq | Liver | 10× Genomics | 1 | 2475 | Integration of droplet-based single nucleus transcriptomics data enabled identification of a small cluster of inactive hepatic stellate cells | [43] |
|  |  | Liver | Drop-seq |  | 1386 |  |  |
|  |  | Liver | 10× Genomics | 6 | 44546 | First to demonstrate the heterogeneity of macrophages associated with liver MDB formation in mice through single-cell resolution | [44] |
| Mouse | ST | Liver | 10× Genomics | 16 | 82,168 | Landscape of hepatocytes and non-parenchymal cells in healthy and NAFLD mouse liver | [45] |
|  | scRNA-seq | Liver | 10× Genomics | 6 | 33,168 | Landscape of intercellular crosstalk in healthy and NASH liver revealed by single-cell secretome gene analysis | [46] |
|  |  | Liver | Bravo automated liquid handling platform (Agilent) | 4 | 1500 | Single-cell spatial reconstruction reveals global division of labor in the mammalian liver | [47] |
|  |  | Liver | 10× Genomics | 4 | 6,000 | Provide evidence for combinatorial effects of diet and anatomic location on regulatory pathways and transcription factors that explain the emergence of disease-associated macrophage phenotypes. | [48] |
|  |  | Liver | Illumina NovaSeq 6000 | 6 | – | TSP1 is significantly expressed in liver nonparenchymal cells and has minimal expression in hepatocytes. | [49] |
|  |  | Liver | Illumina HiSeq-4000 | 8 | 454 | Characterized how the transcriptomic landscape of individual hepatocytes is altered in response to HFD and NAFLD. | [50] |
| Pigs | Drop-seq | Liver | 10× Genomics | – | 3005 | Epigenetically reprogrammed vascular adaptation contributes to liver fibrosis. Targeting of a vascular adaptation node might block maladaptive vascularization to promote liver regeneration in NASH. | [41] |

–: no data; scRNA-seq: single-cell RNA sequencing; snRNA-seq: single-nucleus RNA sequencing; ST: spatial transcriptomics; Drop-Seq: droplet sequencing; Ref.: references; HSC: hematopoietic stem cell; MDB: Mallory-Denk bodies; NAFLD: nonalcoholic fatty liver disease; NASH: nonalcoholic steatohepatitis; TSP1: thrombospondin-1; HFD: high-fat diet.

# Supplementary Table 4. Summary of the application of SCM-Omics and SM-Omics technologies in CVD.

| Species | Sequencing method | Tissues | Platform | Samples | Number of captured cells / Tissue-covered spot | Main findings | Ref. |
| --- | --- | --- | --- | --- | --- | --- | --- |
| Human | scRNA-seq | Heart | Smart-seq2 | 4 | 892 | Defining the earliest step of cardiovascular lineage segregation | [51] |
|  |  | Heart | CEL-seq2 | 5 | 1000 | Single-cell transcriptomics provides insights into hypertrophic cardiomyopathy | [52] |
|  |  | Blood and plaque tissue | 10× Genomics | 15 | 9,490 | Single-cell immune landscape of human atherosclerotic plaques | [53] |
|  |  | Carotid artery | Illumina NextSeq 500 | 6 | 11,019 | VSMC-derived macrophage-like cells were found to undergo a series promotion of lysosome-related and inflammation-related genes. | [54] |
|  |  | Heart | 10× Genomics | 14 | 21,422 | Reveals the cellular landscape underlying cardiac function | [55] |
|  |  | Heart | STRT-seq | 18 | 4,948 | Single-Cell Transcriptome Analysis Maps the Developmental Track of the Human Heart | [56] |
|  | snRNA-seq | Blood | 10× Genomics | 1 | 2,379 | Identify Secretory Factors Promoting Human Hematopoietic Stem Cell Development | [57] |
|  |  | Heart | 10× Genomics | 7 | 287,269 | Transcriptional and Cellular Diversity of the Human Heart | [58] |
|  |  | Heart | 10× Genomics | 14 | 487,106 | Cells of the adult human heart | [59] |
|  | ST | Heart | 10× Genomics | 19 | 3,115 | A Spatiotemporal Organ-Wide Gene Expression and Cell Atlas of the Developing Human Heart | [60] |
|  |  | Aorta | 10× Genomics | 1 | 1,873 | Stanford Type A Aortic Dissection Tissue Section by Spatial Transcriptomics | [61] |
|  | snRNA-seq | Heart | 10× Genomics | 52 | 191,795 | Spatial multi-omic map of human myocardial infarction | [62] |
|  | snATAC-seq |  |  |  | 46,068 |  |  |
|  | ST |  |  |  | 91,517 |  |  |
| Mouse | scRNA-seq | Aorta | 10× Genomics | 10 | 1,138 | Atlas of the immune cell repertoire in mouse atherosclerosis defined by scRNA-seq | [63] |
|  |  | Aorta | 10× Genomics | 10 | 1,226 | Reveals the Transcriptional Landscape and Heterogeneity of Aortic Macrophages in Murine Atherosclerosis | [64] |
|  |  | Brain | Smart-Seq2 | 1 | 3,436 | Constitutes a comprehensive molecular atlas of vascular and vessel-associated cell types in the mouse brain and lung | [65] |
|  |  | Lung |  | 1 | 1,504 |  |  |
|  |  | Heart | 10× Genomics | 9 | 30,000 | Reveals dynamic flux of cardiac stromal, vascular and immune cells in health and injury | [66] |
|  |  | Multiple tissues | 10× Genomics | 11 | 39,182 | Single-Cell Transcriptome Atlas of Murine Endothelial Cells | [67] |
|  |  | Lung | 10× Genomics | 6 | 21,726 | Single-cell RNA sequencing profiling of mouse endothelial cells in response to pulmonary arterial hypertension | [68] |
|  |  | Aorta | – | 9 | 15,288 | Meta-Analysis of Leukocyte Diversity in Atherosclerotic Mouse Aortas | [69] |
|  |  | Aorta | 10× Genomics | 4 | 2198 | Single-cell analysis of fate-mapped macrophages reveals heterogeneity, including stem-like properties | [70] |
|  |  | Aorta | Fluidigm C1 | 13 | – | Describe the use of mass cytometry to define the immune cell composition of murine aortas in mild and more advanced atherosclerosis | [71] |
|  |  | Aorta | 10× Genomics | 2 | 1,784 | Reveals Endothelial Plasticity During Diabetic Atherogenesis | [72] |
|  |  | Aorta | 10× Genomics | 5 | 26,257 | An intersegmental single-cell profile reveals aortic heterogeneity | [73] |
|  | snRNA-seq | Aorta | 10× Genomics | 8 | 2,077 | Identified four subsets of vascular macrophages in atherosclerosis | [74] |
|  | ST | Heart | Illumina NextSeq500 | 1 | – | 3D-cardiomics: A spatial transcriptional atlas of the mammalian heart | [75] |
| Monkey | scRNA-seq | Aortic artery and coronary artery | 10× Genomics | 16 | 7,989 | The single-cell transcriptomic landscape of primate arterial aging was characterized | [76] |
|  |  | 45 tissues | DNBelab C Series | 6 | 1,084,164 | Cell transcriptomic atlas of the non-human primate Macaca fascicularis | [77] |
|  | scRNA-seq | 16 tissues | 10× Genomics | 2 | 174,233 | A reference single-cell regulomic and transcriptomic map of cynomolgus monkeys | [78] |
|  | scATAC-seq |  |  |  | 66,566 |  |  |

–: no data; scRNA-seq: single-cell RNA sequencing; snRNA-seq: single-nucleus RNA sequencing; ST: spatial transcriptomics; snATAC-seq: single-nuclear assay for transposase-accessible chromatin sequencing; scATAC-seq: single-cell assay for transposase-accessible chromatin sequencing; CEL-seq: cell expression by linear amplification and sequencing; STRT-seq: single-cell tagged reverse transcription sequencing; Ref.: references; VSMC: vascular smooth muscle cell; 3D: three-dimensional.

# Supplementary **References**

[1] D. Merrick, A. Sakers, Z. Irgebay, et al., Identification of a mesenchymal progenitor cell hierarchy in adipose tissue, Science 364 (2019).

[2] A.D. Hildreth, F. Ma, Y.Y. Wong, et al., Single-cell sequencing of human white adipose tissue identifies new cell states in health and obesity, Nat Immunol. 22 (2021) 639-653.

[3] J. Vijay, M.F. Gauthier, R.L. Biswell, et al., Single-cell analysis of human adipose tissue identifies depot and disease specific cell types, Nat Metab. 2 (2020) 97-109.

[4] J.R. Acosta, S. Joost, K. Karlsson, et al., Single cell transcriptomics suggest that human adipocyte progenitor cells constitute a homogeneous cell population, Stem Cell Res Ther. 8 (2017) 250.

[5] M.P. Emont, C. Jacobs, A.L. Essene, et al., A single-cell atlas of human and mouse white adipose tissue, Nature 603 (2022) 926-933.

[6] W. Sun, H. Dong, M. Balaz, et al., snRNA-seq reveals a subpopulation of adipocytes that regulates thermogenesis, Nature 587 (2020) 98-102.

[7] K.L. Whytock, Y. Sun, A. Divoux, et al., Single cell full-length transcriptome of human subcutaneous adipose tissue reveals unique and heterogeneous cell populations, iScience 25 (2022) 104772.

[8] J. Backdahl, L. Franzen, L. Massier, et al., Spatial mapping reveals human adipocyte subpopulations with distinct sensitivities to insulin, Cell Metab. 33 (2021) 1869-1882 e1866.

[9] C. Hepler, B. Shan, Q. Zhang, et al., Identification of functionally distinct fibro-inflammatory and adipogenic stromal subpopulations in visceral adipose tissue of adult mice, Elife 7 (2018).

[10] W. Gu, W.N. Nowak, Y. Xie, et al., Single-Cell RNA-Sequencing and Metabolomics Analyses Reveal the Contribution of Perivascular Adipose Tissue Stem Cells to Vascular Remodeling, Arterioscler Thromb Vasc Biol. 39 (2019) 2049-2066.

[11] R.B. Burl, V.D. Ramseyer, E.A. Rondini, et al., Deconstructing Adipogenesis Induced by beta3-Adrenergic Receptor Activation with Single-Cell Expression Profiling, Cell Metab. 28 (2018) 300-309 e304.

[12] D.S. Cho, B. Lee, J.D. Doles, Refining the adipose progenitor cell landscape in healthy and obese visceral adipose tissue using single-cell gene expression profiling, Life Sci Alliance 2 (2019).

[13] P.C. Schwalie, H. Dong, M. Zachara, et al., A stromal cell population that inhibits adipogenesis in mammalian fat depots, Nature 559 (2018) 103-108.

[14] P. Rajbhandari, D. Arneson, S.K. Hart, et al., Single cell analysis reveals immune cell-adipocyte crosstalk regulating the transcription of thermogenic adipocytes, Elife 8 (2019).

[15] A.K. Sarvari, E.L. Van Hauwaert, L.K. Markussen, et al., Plasticity of Epididymal Adipose Tissue in Response to Diet-Induced Obesity at Single-Nucleus Resolution, Cell Metab. 33 (2021) 437-453 e435.

[16] R. Dorajoo, Y. Ali, V.S.Y. Tay, et al., Single-cell transcriptomics of East-Asian pancreatic islets cells, Sci Rep. 7 (2017) 5024.

[17] Y.J. Wang, J. Schug, K.J. Won, et al., Single-Cell Transcriptomics of the Human Endocrine Pancreas, Diabetes 65 (2016) 3028-3038.

[18] M.J. Muraro, G. Dharmadhikari, D. Grun, et al., A Single-Cell Transcriptome Atlas of the Human Pancreas, Cell Syst. 3 (2016) 385-394 e383.

[19] A. Segerstolpe, A. Palasantza, P. Eliasson, et al., Single-Cell Transcriptome Profiling of Human Pancreatic Islets in Health and Type 2 Diabetes, Cell Metab. 24 (2016) 593-607.

[20] J. Li, J. Klughammer, M. Farlik, et al., Single-cell transcriptomes reveal characteristic features of human pancreatic islet cell types, EMBO Rep. 17 (2016) 178-187.

[21] N. Lawlor, J. George, M. Bolisetty, et al., Single-cell transcriptomes identify human islet cell signatures and reveal cell-type-specific expression changes in type 2 diabetes, Genome Res. 27 (2017) 208-222.

[22] A.N. Lam, M.A. Aksit, B. Vecchio-Pagan, et al., Increased expression of anion transporter SLC26A9 delays diabetes onset in cystic fibrosis, J Clin Invest. 130 (2020) 272-286.

[23] P.S. Linsley, F. Barahmand-Pour-Whitman, E. Balmas, et al., Autoreactive T cell receptors with shared germline-like alpha chains in type 1 diabetes, JCI Insight. 6 (2021).

[24] M. Saikia, M.M. Holter, L.R. Donahue, et al., GLP-1 receptor signaling increases PCSK1 and beta cell features in human alpha cells, JCI Insight. 6 (2021).

[25] C. Su, L. Gao, C.L. May, et al., 3D chromatin maps of the human pancreas reveal lineage-specific regulatory architecture of T2D risk, Cell Metab. 34 (2022) 1394-1409 e1394.

[26] G. Basile, S. Kahraman, E. Dirice, et al., Using single-nucleus RNA-sequencing to interrogate transcriptomic profiles of archived human pancreatic islets, Genome Med. 13 (2021) 128.

[27] D.E. Stanescu, R. Yu, K.J. Won, et al., Single cell transcriptomic profiling of mouse pancreatic progenitors, Physiol Genomics 49 (2017) 105-114.

[28] C. Zeng, F. Mulas, Y. Sui, et al., Pseudotemporal Ordering of Single Cells Reveals Metabolic Control of Postnatal beta Cell Proliferation, Cell Metab. 25 (2017) 1160-1175 e1111.

[29] N. Sharon, R. Chawla, J. Mueller, et al., A Peninsular Structure Coordinates Asynchronous Differentiation with Morphogenesis to Generate Pancreatic Islets, Cell 176 (2019) 790-804 e713.

[30] A.M. Hendley, A.A. Rao, L. Leonhardt, et al., Single-cell transcriptome analysis defines heterogeneity of the murine pancreatic ductal tree, Elife 10 (2021).

[31] Y. Xin, J. Kim, M. Ni, et al., Use of the Fluidigm C1 platform for RNA sequencing of single mouse pancreatic islet cells, Proc Natl Acad Sci U S A. 113 (2016) 3293-3298.

[32] N.A.J. Krentz, M.Y.Y. Lee, E.E. Xu, et al., Single-Cell Transcriptome Profiling of Mouse and hESC-Derived Pancreatic Progenitors, Stem Cell Reports 11 (2018) 1551-1564.

[33] D. Wang, J. Wang, L. Bai, et al., Long-Term Expansion of Pancreatic Islet Organoids from Resident Procr(+) Progenitors, Cell 180 (2020) 1198-1211 e1119.

[34] S. Sachs, A. Bastidas-Ponce, S. Tritschler, et al., Targeted pharmacological therapy restores beta-cell function for diabetes remission, Nat Metab. 2 (2020) 192-209.

[35] T. Fukaishi, Y. Nakagawa, A. Fukunaka, et al., Characterisation of Ppy-lineage cells clarifies the functional heterogeneity of pancreatic beta cells in mice, Diabetologia 64 (2021) 2803-2816.

[36] P.N. Zakharov, H. Hu, X. Wan, et al., Single-cell RNA sequencing of murine islets shows high cellular complexity at all stages of autoimmune diabetes, J Exp Med. 217 (2020).

[37] P. Chen, F. Yao, Y. Lu, et al., Single-Cell Landscape of Mouse Islet Allograft and Syngeneic Graft, Front Immunol. 13 (2022) 853349.

[38] E. Duvall, C.M. Benitez, K. Tellez, et al., Single-cell transcriptome and accessible chromatin dynamics during endocrine pancreas development, Proc Natl Acad Sci U S A. 119 (2022) e2201267119.

[39] Z.Y. Wang, A. Keogh, A. Waldt, et al., Single-cell and bulk transcriptomics of the liver reveals potential targets of NASH with fibrosis, Sci Rep. 11 (2021) 19396.

[40] S.A. MacParland, J.C. Liu, X.Z. Ma, et al., Single cell RNA sequencing of human liver reveals distinct intrahepatic macrophage populations, Nat Commun. 9 (2018) 4383.

[41] H. Zhang, Y. Ma, X. Cheng, et al., Targeting epigenetically maladapted vascular niche alleviates liver fibrosis in nonalcoholic steatohepatitis, Sci Transl Med. 13 (2021) eabd1206.

[42] T.S. Andrews, J. Atif, J.C. Liu, et al., Single-Cell, Single-Nucleus, and Spatial RNA Sequencing of the Human Liver Identifies Cholangiocyte and Mesenchymal Heterogeneity, Hepatol Commun. 6 (2022) 821-840.

[43] K. Diamanti, J.S. Inda Diaz, A. Raine, et al., Single nucleus transcriptomics data integration recapitulates the major cell types in human liver, Hepatol Res. 51 (2021) 233-238.

[44] R. Zhang, B. Zhong, J. He, et al., Single-cell transcriptomes identifies characteristic features of mouse macrophages in liver Mallory-Denk bodies formation, Exp Mol Pathol. 127 (2022) 104811.

[45] Q. Su, S.Y. Kim, F. Adewale, et al., Single-cell RNA transcriptome landscape of hepatocytes and non-parenchymal cells in healthy and NAFLD mouse liver, iScience 24 (2021) 103233.

[46] X. Xiong, H. Kuang, S. Ansari, et al., Landscape of Intercellular Crosstalk in Healthy and NASH Liver Revealed by Single-Cell Secretome Gene Analysis, Mol Cell 75 (2019) 644-660 e645.

[47] K.B. Halpern, R. Shenhav, O. Matcovitch-Natan, et al., Single-cell spatial reconstruction reveals global division of labour in the mammalian liver, Nature 542 (2017) 352-356.

[48] J.S. Seidman, T.D. Troutman, M. Sakai, et al., Niche-Specific Reprogramming of Epigenetic Landscapes Drives Myeloid Cell Diversity in Nonalcoholic Steatohepatitis, Immunity 52 (2020) 1057-1074 e1057.

[49] T. Gwag, R.G. Reddy Mooli, D. Li, et al., Macrophage-derived thrombospondin 1 promotes obesity-associated non-alcoholic fatty liver disease, JHEP Rep. 3 (2021) 100193.

[50] S.R. Park, C.S. Cho, J. Xi, et al., Holistic characterization of single-hepatocyte transcriptome responses to high-fat diet, Am J Physiol Endocrinol Metab. 320 (2021) E244-E258.

[51] F. Lescroart, X. Wang, X. Lin, et al., Defining the earliest step of cardiovascular lineage segregation by single-cell RNA-seq, Science 359 (2018) 1177-1181.

[52] M. Wehrens, A.E. de Leeuw, M. Wright-Clark, et al., Single-cell transcriptomics provides insights into hypertrophic cardiomyopathy, Cell Rep. 39 (2022) 110809.

[53] D.M. Fernandez, A.H. Rahman, N.F. Fernandez, et al., Single-cell immune landscape of human atherosclerotic plaques, Nat Med. 25 (2019) 1576-1588.

[54] Z. Zhang, J. Huang, Y. Wang, et al., Transcriptome analysis revealed a two-step transformation of vascular smooth muscle cells to macrophage-like cells, Atherosclerosis 346 (2022) 26-35.

[55] L. Wang, P. Yu, B. Zhou, et al., Single-cell reconstruction of the adult human heart during heart failure and recovery reveals the cellular landscape underlying cardiac function, Nat Cell Biol. 22 (2020) 108-119.

[56] Y. Cui, Y. Zheng, X. Liu, et al., Single-Cell Transcriptome Analysis Maps the Developmental Track of the Human Heart, Cell Rep. 26 (2019) 1934-1950 e1935.

[57] E.I. Crosse, S. Gordon-Keylock, S. Rybtsov, et al., Multi-layered Spatial Transcriptomics Identify Secretory Factors Promoting Human Hematopoietic Stem Cell Development, Cell Stem Cell 27 (2020) 822-839 e828.

[58] N.R. Tucker, M. Chaffin, S.J. Fleming, et al., Transcriptional and Cellular Diversity of the Human Heart, Circulation 142 (2020) 466-482.

[59] M. Litvinukova, C. Talavera-Lopez, H. Maatz, et al., Cells of the adult human heart, Nature 588 (2020) 466-472.

[60] M. Asp, S. Giacomello, L. Larsson, et al., A Spatiotemporal Organ-Wide Gene Expression and Cell Atlas of the Developing Human Heart, Cell 179 (2019) 1647-1660 e1619.

[61] Y.H. Li, Y. Cao, F. Liu, et al., Visualization and Analysis of Gene Expression in Stanford Type A Aortic Dissection Tissue Section by Spatial Transcriptomics, Front Genet. 12 (2021) 698124.

[62] C. Kuppe, R.O. Ramirez Flores, Z. Li, et al., Spatial multi-omic map of human myocardial infarction, Nature 608 (2022) 766-777.

[63] H. Winkels, E. Ehinger, M. Vassallo, et al., Atlas of the Immune Cell Repertoire in Mouse Atherosclerosis Defined by Single-Cell RNA-Sequencing and Mass Cytometry, Circ Res. 122 (2018) 1675-1688.

[64] C. Cochain, E. Vafadarnejad, P. Arampatzi, et al., Single-Cell RNA-Seq Reveals the Transcriptional Landscape and Heterogeneity of Aortic Macrophages in Murine Atherosclerosis, Circ Res. 122 (2018) 1661-1674.

[65] L. He, M. Vanlandewijck, M.A. Mae, et al., Single-cell RNA sequencing of mouse brain and lung vascular and vessel-associated cell types, Sci Data. 5 (2018) 180160.

[66] N. Farbehi, R. Patrick, A. Dorison, et al., Single-cell expression profiling reveals dynamic flux of cardiac stromal, vascular and immune cells in health and injury, Elife 8 (2019).

[67] J. Kalucka, L. de Rooij, J. Goveia, et al., Single-Cell Transcriptome Atlas of Murine Endothelial Cells, Cell 180 (2020) 764-779 e720.

[68] J. Rodor, S.H. Chen, J.P. Scanlon, et al., Single-cell RNA sequencing profiling of mouse endothelial cells in response to pulmonary arterial hypertension, Cardiovasc Res. 118 (2022) 2519-2534.

[69] A. Zernecke, H. Winkels, C. Cochain, et al., Meta-Analysis of Leukocyte Diversity in Atherosclerotic Mouse Aortas, Circ Res. 127 (2020) 402-426.

[70] J.D. Lin, H. Nishi, J. Poles, et al., Single-cell analysis of fate-mapped macrophages reveals heterogeneity, including stem-like properties, during atherosclerosis progression and regression, JCI Insight. 4 (2019).

[71] J.E. Cole, I. Park, D.J. Ahern, et al., Immune cell census in murine atherosclerosis: cytometry by time of flight illuminates vascular myeloid cell diversity, Cardiovasc Res. 114 (2018) 1360-1371.

[72] G. Zhao, H. Lu, Y. Liu, et al., Single-Cell Transcriptomics Reveals Endothelial Plasticity During Diabetic Atherogenesis, Front Cell Dev Biol. 9 (2021) 689469.

[73] L. Yu, J. Zhang, A. Gao, et al., An intersegmental single-cell profile reveals aortic heterogeneity and identifies a novel Malat1(+) vascular smooth muscle subtype involved in abdominal aortic aneurysm formation, Signal Transduct Target Ther. 7 (2022) 125.

[74] S. McArdle, K. Buscher, Y. Ghosheh, et al., Migratory and Dancing Macrophage Subsets in Atherosclerotic Lesions, Circ Res. 125 (2019) 1038-1051.

[75] M. Mohenska, N.M. Tan, A. Tokolyi, et al., 3D-cardiomics: A spatial transcriptional atlas of the mammalian heart, J Mol Cell Cardiol. 163 (2022) 20-32.

[76] W. Zhang, S. Zhang, P. Yan, et al., A single-cell transcriptomic landscape of primate arterial aging, Nat Commun. 11 (2020) 2202.

[77] L. Han, X. Wei, C. Liu, et al., Cell transcriptomic atlas of the non-human primate Macaca fascicularis, Nature 604 (2022) 723-731.

[78] J. Qu, F. Yang, T. Zhu, et al., A reference single-cell regulomic and transcriptomic map of cynomolgus monkeys, Nat Commun. 13 (2022) 4069.
